# Hearing and vision care provided to older people residing in care homes: a cross-sectional survey of care home staff

**DOI:** 10.1186/s12877-020-01959-0

**Published:** 2021-01-08

**Authors:** Wendy Andrusjak, Ana Barbosa, Gail Mountain

**Affiliations:** Centre for Applied Dementia Studies, Faculty of Health Studies, University of Bradford, Dementia, Richmond Road, Bradford, BD7 1DP UK

**Keywords:** Nursing, Care home, Hearing, Vision, Sensory, Nursing home, Long term care

## Abstract

**Background:**

Hearing and vision loss in older people has been proven to affect physical and mental health and increase the speed of cognitive decline. Studies have demonstrated that certain practices and improved staff knowledge increase the effective care of residents’ ears and eyes, yet it is not known which practices are being implemented in care homes. This study aimed to identify the gaps in staff knowledge regarding hearing and vision difficulties in older residents, and which practices known to improve ear and eye care in older care home residents are not commonly implemented in care homes in England.

**Methods:**

This study used a cross-sectional survey design. Survey questions were informed by the existing literature and were focused on practices, staff knowledge, and other aspects that have shown to affect residents’ hearing and vision care. A convenience sample of care home staff were recruited from care homes across England between November 2018 and February 2019 via email and in paper format. Descriptive statistics and Chi-Square analysis were applied to identify the factors influencing the care being provided to care home residents.

**Results:**

A total of 400 care home staff responded from 74 care homes. The results revealed that less than half of staff respondents reported to use screening tools to identify hearing (46%) and vision impairments (43.8%); that care homes rarely have access to other assistive devices for hearing (16%) and vision loss (23.8%), and that audiology services do not regularly assess care home residents (46.8%). A majority of staff who responded were not confident in ear and eye care. Responses were found to be influenced by the respondents’ job role, length of time working in care homes and also the care home type. Findings confirmed a lack of standardised practice and the importance of shared communication for promulgation of best practice.

**Conclusion:**

This study has identified that some practices known to facilitate ear and eye care are not commonly applied in a sample of English care homes. It has also shown that care home staff knowledge of ear and eye care is inconsistent. The information derived from this survey can be used to inform guidelines for best practice and inform needs for future research.

**Supplementary Information:**

The online version contains supplementary material available at 10.1186/s12877-020-01959-0.

## Background

Hearing and vision loss in older adults is challenging for them and can also affect other health related outcomes, including an increased rate of cognitive decline, increased risk of falls and reduced quality of life [1–3]. If care homes are to improve such outcomes then effective hearing and vision care should be provided to older residents. This is likely to be reliant upon good staff training on how to provide routine care, identify problems being experiences by residents, instigate effective aid management and ensure provision of regular hearing and vision tests. However, it has been found that there are many barriers to providing quality ear and eye care in care homes, including staff knowledge, residents cognition, management of assistive devices and the environment [4]. To overcome these requires understanding of current practice, including the barriers to implementing best practice and how they might be overcome.

In England there are around 400,000 older people residing in care homes [5], with 75% of these individuals estimated to be living with some degree of hearing loss [6] and around 50% with some degree of vision loss [7]. However, these sensory impairments often go undetected or are poorly managed, resulting in residents living with limited hearing and vision capabilities [8]. The consequences can significantly negatively affect residents’ quality of life; this includes physical health, mental health, and opportunities for social interaction [3, 9, 10]. Despite these acknowledged impacts there is still a lack of focus on sensory impairment identification and management in older people in care homes. Evidence suggests that this can be due to the common perception by care home staff and residents that hearing and vision loss are normal aspects associated with ageing [8]; This contributes towards hearing and vision loss becoming hidden health issues within this population.

A recent review has found that there are other barriers in care homes that contribute to the poor identification and management of hearing and vision loss in residents [4]. These include a lack of care home staff knowledge, poor management of assistive aids (e.g. hearing aids and glasses), limited access to specialist services such as audiologists and optometrists, under-use of screening tools, an unsuitable care home environment, and residents’ reduced cognitive capabilities [4]. A number of studies aimed to increase knowledge in staff using methods such as educational videotapes [11], workshops [12], or by attaching instructions about insertion and maintenance of hearing aids to the inside of residents’ wardrobes [13]. Training residents to manage their own devices has also been applied in one identified study [14]. Whilst these practices were reportedly beneficial in increasing staff and residents’ knowledge, how and why these have been effective is largely unknown. Furthermore, screening tools such as the Nursing Home Handicap Hearing Index [15] test and the Minimum Data Set [16] have been used by staff to assess residents’ sensory difficulties, but the review found that use of such instruments was problematic in practice [4]. Most research to date has focused upon highlighting the barriers and facilitators in care homes, with minimal research evaluating interventions aimed at improving current best practice [4].

Other evidence also reveals reluctance among residents to use their prescribed assistive aids and reduced ability to manage their own aids without assistance [11]. Whilst the emphasis is mainly on staff and professionals providing the required treatment, care and support, family members can also assist with management of ear and eye care [17, 18]. Hearing and vision charitable organisations have produced guidelines in effort to overcome some of the barriers to best care [6, 19]. However, more evidence is required to underpin such guidance.

Whilst it is important to have a range of practices available to effectively support residents with sensory impairments, it is also important to ensure that care home staff are readily aware of best practice and what might be practically implemented in their place of work [20]. They must also be sufficiently knowledgeable regarding what interventions they might readily implement and how to do this [21]. Improved care home staff knowledge of different hearing and vision conditions might assist with identifying what each resident specifically struggles with in everyday life, and thus what practices would be most beneficial. In addition, staff should know how to correctly clean and maintain hearing aids and glasses to ensure they are functioning correctly and can therefore be used to best effect [6, 19].

It is important for staff to understand how to effectively and efficiently improve current hearing and vision practices in care homes. However, the everyday practices used in care homes are unknown. The aim of the study described in this paper was to identify the practices care homes in England currently use and the level of knowledge staff have when caring for residents’ ears and eyes. All care homes in England have their quality of care rated by the Care Quality Commission. We have included care homes with different ratings as this will ensure the study includes the different standards of care homes across the country. By highlighting the practices most and least used and the gaps in staff knowledge, we can improve ear and eye care in this vulnerable population. To address the study purpose, we aimed to:

1. To identify which practices are currently being used in care homes across England to care for older residents’ hearing and vision difficulties
2. To assess care home staff knowledge of hearing and vision care in older residents
3. To identify any relationships between the characteristics of staff and the care homes they work in, staff knowledge of ear and eye care, and use of different practices and interventions.

## Methods

### Design

A cross-sectional survey methodology was used to address the study aims. The survey used was designed by the researchers to reflect the factors identified in the literature as affecting ear and eye care [4]. This was further developed with an advisory group to ensure it was relatable to the intended population.

### Participants and setting

The survey was designed for completion by all staff either directly responsible for and/or in regular contact with residents such as health care assistants, trained nurses, care home managers and activity coordinators. Care homes to be surveyed included both nursing homes (which are homes that provide 24-h nursing care to residents) and residential homes (which provide assistance to residents but do not generally include nursing care). All care homes that care for older people were deemed eligible to take part. An email was sent to care home managers who distributed the survey to eligible staff. A convenience sample of care home staff members who had regular contact with older care home residents was recruited. Given the limited resources and time available, a convenience method of recruitment, whereby any eligible participant was approached for participation, was used to obtain the largest sample possible in a short time period. To be included in the study, care home staff had to be working in a care home for adults over 65 years old and have regular responsibilities for resident care. They also had to be able to read and write in English.

### Survey development and piloting

Survey questions were informed by the existing literature and were focused on practices, staff knowledge, and other aspects that have shown to affect residents’ sensory care such as residents’ willingness to use aids and their ability to care for their aids themselves [4]. The survey (Additional file 1) was designed in three sections: the first section requested details of the respondent and the care home they work in, this included the type of care home where the respondent worked they were able to select more than one type. The second section queried the hearing practices used in the care home and staff knowledge of ear care, and the third section was concerned with vision practices used and staff knowledge of eye care. The 41 questions in the survey included were derived and informed by the literature: three questions to determine if eligibility criteria were met; four questions requesting brief demographic details of the respondent; 33 multiple choice questions about hearing and vision care in the home the respondent worked in; One free text question requested details of other services that contribute to the care of residents hearing and vision. (Additional file 1). In addition, postcodes of respondents’ care home were collected using a free text question at the beginning of the survey to assess the Care Quality Commission (CQC) rating of the home (which assesses the standard of care homes across England); this was to gain knowledge of the ratio of care homes requiring improvement, those classed as good, and those as outstanding.

The survey was designed using the JISC online survey website [22]. Information for respondents about the overall study, ethics and data management was included in the first page of the survey (Additional file 1). A question was included at the end of this page for the participant to confirm that they had read the information and agreed to voluntarily take part in the survey, which if they continued and completed the survey it was taken as informed consent. All questions on the electronic survey were required to be answered for progression to the next question to be possible.

The draft survey designed from the literature was reviewed and refined following consultation with a study advisory group. This advisory group comprised of two health care assistants, an activities coordinator, the head of care home services and a care home research nurse who works for the Enabling Research in Care Homes (ENRICH) network. The ENRICH network is a resource that supports social care research across England, encouraging care home participation in research and assisting in the design of studies to best suit this population. A face to face meeting was convened with the group who were asked to complete the survey in situ. They were also asked to consider the wording of the cover letter and the survey questions, the appropriateness of the multiple choices given for each question and the proposed modes of survey distribution. Input of the advisory group led to changes to the wording of some questions and changes to the cover letter so that all care staff in regular contact with residents might readily understand it. In response to suggestions, additional response options were included for certain questions (e.g. refer to appended questionnaire questions 19 and 36); the need for the survey to be made available in both paper and electronic versions was emphasised.

The survey was firstly piloted in two nursing homes in the North of England which were recognised as being ‘research ready’ by the ENRICH team. The two nursing homes received the survey firstly via email and then were also sent a paper version so that both presentations were piloted. Each of the testing aspects, such as the survey design, methods of distribution and data collection with the successful return of four paper and four electronic survey copies, were confirmed and deemed feasible.

### Data collection

The study gained UK National Institute for Health Research (NIHR) portfolio status, which is a service provided by the National Health Service (NHS) to support studies that are of benefit to the UK NHS. This enabled the survey to be nationally distributed to care homes via relevant UK research networks, (the ENRICH network and the Clinical Research Network), thereby reaching a wider population. The web link to the survey was distributed to care home managers via email between November 2018 and February 2019 by the ENRICH network and the national Clinical Research Network. Additionally, social media and conferences were used to advertise the survey to other care homes with an internet link to the survey included in the information provided about the study. No incentives were given to care homes to encourage participation other than highlighting how this research can inform future practice.

Both electronic and paper methods were used for the distribution of the survey (Additional file 1). The survey was first distributed to care home managers across the country via email, with a link to the online survey. Managers were then requested to distribute the survey to their staff who met the inclusion criteria and were interested in taking part. The option for paper copies was also offered in the same Email. Paper copies were printed and sent to care homes who requested them with a pre-paid return envelope.

### Data analysis

Full data sets were obtained from respondents. The survey responses were inputted onto statistical analysis software SPSS version 25. Descriptive statistics were firstly produced to give percentage responses to each of the questions. Chi-Square analyses were then conducted to identify any relationships between the sociodemographic profiles of respondents and care home practices. As many tests were to be performed (16 tests for vision and 15 about hearing), the Bonferroni adjustment was used to reduce the likelihood of false findings of significance and a *p*-value of 0.000 was applied to determine any significant results [23].

### Ethical considerations

University ethical approval was obtained for this study (Registration number: EC25216). The final survey design was produced using Jisc online surveys website [22]. This site is UK based and adheres to all General Data Protection Guidelines (GDPR) enforced in the UK, ensuring participants’ data is effectively and appropriately managed. All data was held securely to meet regulations. Care home postcodes are not identified or reported in this publication. As per GDPR guidelines, this information has been stored securely on a password protected computer that is only accessed by the research team.

## Results

A total of 400 survey questionnaires were returned from staff working in 74 care homes across England.

### Characteristics of care home and respondents

Due to some respondents not completing the postcode free text question accurately, only 66 of the 74 responding care homes could be identified and their CQC rating found; 15 required improvement (22.7%), 44 were reported as good (66.6%), and seven were outstanding (10.6%). The responses of staff from each home were collated to determine the number of respondents from each care home, this ranged from one person to 36 people, with 26 care homes having five or more respondents.

Analysis found that many respondents from the same care home reported different care home types for their home. Due to these disparities, all care home classifications were then checked against the care home manager’s response and for those who provided a valid postcode the care home website was reviewed to provide a more accurate and standardised record of what type of care home it was.

Survey respondents were most commonly working in nursing homes (180/ 45%), followed by residential care homes (144/ 36%). Some respondents also noted they worked in a care home caring especially for those living with dementia (16/ 4%). Two dual-registered groups were also formed as some respondents commonly reported their home had both nursing and residential units (44/ 11%), or that their home was a residential unit but specifically caring for those living with dementia (12/ 3%). In addition, some noted their work place as ‘other’ (4/ 1%), which were settings that the respondent felt did not fit into any of these other categories.

Respondents job roles included health care assistants (who provide personal cares/assistance to residents) (210/ 52.5%), care home managers (65/ 16.25%), qualified nurses (56/ 14%), activities coordinators (14/ 3.5%) and ‘other’ for those who did not consider that they were in any of the other role options (55/ 13.8%). Elaboration on other job roles was enabled through a free text option. The role ‘other’ mainly included those classifying themselves as senior health care assistants, but also included catering staff.

Size of the care home was based on the amount of residents the care home could accommodate, and therefore related to resident capacity. Smaller care homes were considered to be those with less than 30 residents (118/ 29.5%), medium sized care homes as those with a resident capacity of between 31 and 60 (142/ 35.5%) and larger care homes being able to accommodate over 60 residents (140/ 35%). Information on the length of time the respondent had worked in care homes was also collected; less than 2 years (78/ 19.5%), 2–5 years (122/ 30.5%), 5–10 years (62/15.5%), and over 10 years (138/ 34.5%).

There was a significant correlation between job role and length of time that respondents had worked in care homes (*P* = 0.000), with care home managers reporting the longest length of service and health care assistants the least. A significant relationship was also found between care home type and job role (P = 0.000), with a greater proportion of respondents from nursing homes being qualified nurses, and a greater proportion of those classifying themselves as ‘other’ being from residential homes.

**Fig. 1:**
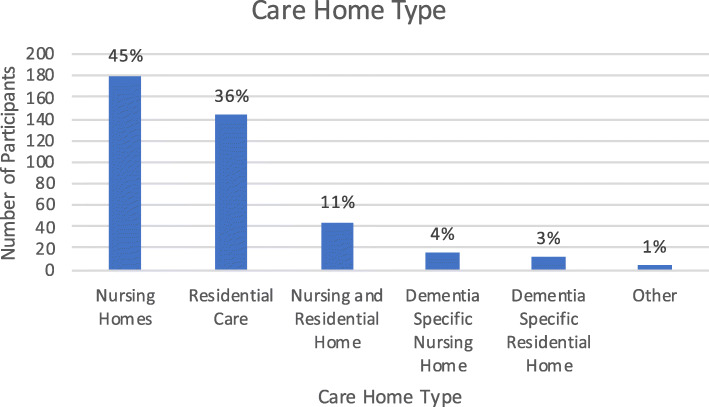
Care Home Type where the Respondents Worked

**Fig. 2:**
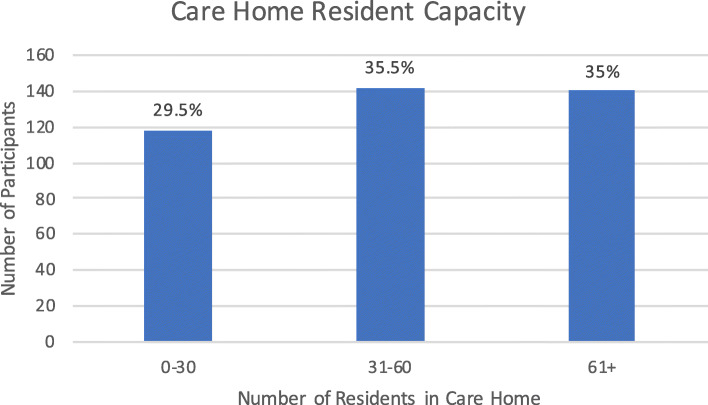
Number of Residents in the Respondents Care Home

**Fig. 3:**
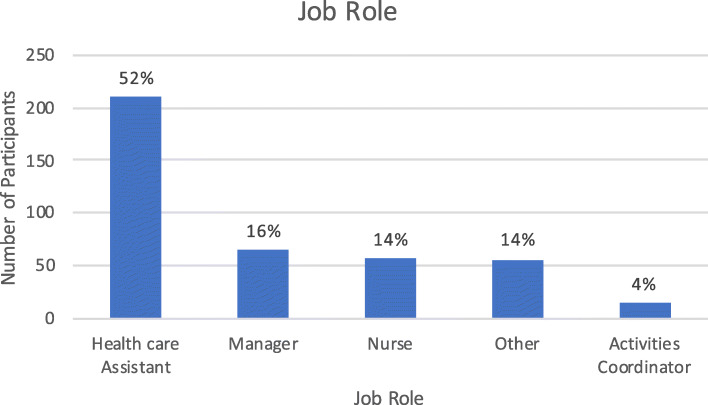
Job Role of Respondent

**Fig. 4:**
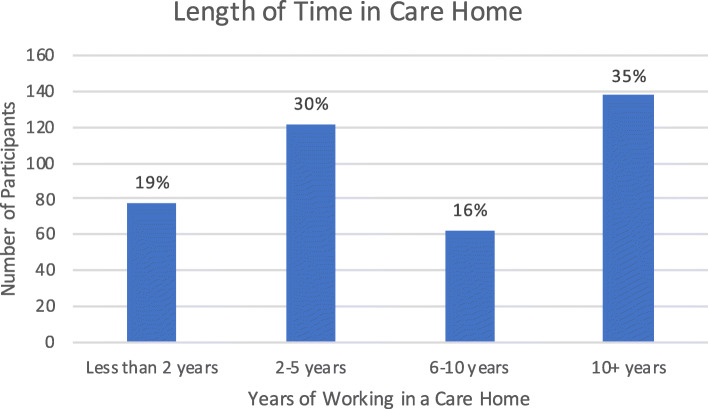
Length of Time the Respondent had worked in Care Homes

The figures below show the number of responses for the different types of care home the respondent stated that they worked in (Fig. 1), the size of the care home (Fig. 2), their reported job role (Fig. 3), and the length of the time they had worked in care homes (Fig. 4).

### Care home practices and staff knowledge

Table 1 shows the percentage of responses regarding assessment and treatment of hearing loss and vision loss. The majority of participants reported that both hearing (91.3%) and vision impairments (93.5%) are recorded in residents’ care plans, and that hearing aids (84%) and glasses (91.8%) are checked/cleaned regularly by staff. In addition, a majority reported that their care home has made suitable environment adaptations such as quiet places for residents (79.5%), that the care home they work in is well lit (88.3%), and that specific adaptions to the environment (such as contrasting colours for signs) exist (67.8%). In addition, the majority said that annual vision check-ups by optometrists are conducted in the home (85.3%).

**Table 1:** Hearing and vision practices reportedly implemented in care homes (*n* = 400)

Practices (%/n)	Yes	No	Not sure	Sometimes	When residents bring their own
*Hearing*					
Care plan (Hearing)	91.3 (365)	0.3 (1)	2 (8)	6.5 (26)	
Hearing Aids checked regularly	84 (336)	5 (20)	11 (44)		
Quiet places	79.5 (318)	14.2 (57)	6.3 (25)		
Annual hearing check-ups	46.8 (187)	25 (100)	28.2 (113)		
Hearing Screening Tools	16 (64)	46 (184)	38 (152)		
Access to other hearing devices	15 (60)	29.8 (119)	18.8 (75)		36.5 (146)
*Vision*					
Care plan (Vision)	93.5 (374)	0.5 (2)	2.5 (10)	3.5 (14)	
Glasses cleaned regularly	91.8 (367)	4.5 (18)	3.8 (15)		
Well-lit rooms	88.3 (353)	5.3 (21)	6.5 (26)		
Annual vision check-ups	85.3 (341)	2.8 (11)	12 (48)		
Adaptations to environment (Vision)	67.8 (271)	21.8 (87)	10.5 (42)		
Glasses labelled with owner’s name	48.8 (195)	7.2 (29)	4 (16)	40 (160)	
Access to other vision devices	45 (180)	8.3 (33)	13.5 (54)		33.3 (133)
Vision Screening Tools	23.8 (95)	43.8 (175)	32.5 (130)		

However, some hearing and vision practices were less commonly implemented. A total of 46% of participants stated that they did not use screening tools for hearing assessment; and 43.8% not using vision screening tools. Assistive devices for hearing were only reported by 16%; and only 23.8% of care homes provided other assistive devices for vision part from prescribed glasses (i.e. magnifying glasses). Less than half stated that annual hearing check-ups are conducted by audiologists (46.8%), and that all residents’ glasses are labelled (48.8%).

Just over a quarter (103 out of the 400 respondents) reported that all of their residents are willing to use their hearing aids (25.8%), and only 83 stated that their residents are able to take care of their own aids (20.8%). Furthermore, the majority of all respondents reported that some assistance in caring for residents’ hearing problems is provided by family members (96%). Only 118 out of the 400 respondents reported that all residents are willing to use their glasses (29.5%), with 359 stating that family members provide assistance with this (89.8%).

A free text question asked about other practices used in the care home to assist with the identification and management of hearing and vision difficulties apart from those that had been listed. Additional hearing practices included using sign language professionals, nurses carrying out ear syringing, creating links with relevant charities, use of communications devices such as IPads, and staff training. Examples given of additional vision practices were contacts with relevant charities, input from a variety of outside professionals, volunteers, one on one care, visits from professional services that provide eye care, and staff assistance and training.

Table 2 shows the percentage of respondents who considered themselves to be confident in their knowledge of different aspects of hearing and vision care. For all questions about staff knowledge, over 50% reported being confident to some extent in their knowledge of a particular aspect of hearing and vision care. The majority of respondents strongly agreed that they were confident in cleaning glasses (57.5%) and communicating with the hearing (63.5%) or visually impaired residents (70.5%). However, less than 25% strongly agreed that they were confident in recognising various hearing (17.5%) and vision conditions (8.3%), assessing whether a resident has a hearing (24.8%) or vision impairment (14%), assessing hearing (21.5%) or vision impairment in the cognitively impaired (14.5%) and cleaning hearing aids (23.8%).

**Table 2:** Staff knowledge of hearing and vision care (*n* = 400)

Knowledge (%/n)	Strongly Agree	Agree	Disagree	Strongly Disagree
*Hearing*				
Communicating with the hearing impaired	63.5 (254)	30 (120)	5.5 (22)	1 (4)
Assessing whether a resident has a hearing impairment	24.8 (99)	60 (240)	13.3 (53)	2 (8)
Cleaning hearing aids	23.8 (95)	54 (216)	18.5 (74)	3.8 (15)
Assessing hearing impairment in the cognitively impaired	21.5 (86)	55 (220)	21.3 (85)	2.3 (9)
Knowing the difference between hearing conditions	17.5 (70)	56 (224)	24.5 (98)	2 (8)
*Vision*				
Communicating with the vision impaired	70.5 (282)	25.8 (103)	3.5 (14)	0.3 (1)
Cleaning glasses	57.5 (230)	39.5 (158)	2.3 (9)	0.8 (3)
Assessing vision impairment in the cognitively impaired	14.5 (58)	51.7 (207)	30.8 (123)	3 (12)
Assessing whether a resident has a vision impairment	14 (56)	59.3 (237)	25 (100)	1.8 (7)
Knowing the difference between vision conditions	8.3 (33)	43 (172)	42 (168)	6.8 (27)

When asked whether the respondent would like more information regarding how to effectively identify and manage hearing and vision loss, 89.5% agreed that they would like more information on hearing loss, and 85.3% agreed they would like to receive this for vision loss.

For all homes with five or more staff who responded (*n* = 26), a range of responses were provided about the practices the care home was implementing. Whilst some working in the same home agreed that certain practices took place in their care home, others from that same care home were unsure or disagreed; This highlights uncertainty amongst staff regarding the practices the care homes they worked in were actually implementing.

### Relationship between hearing and vision care and participant characteristics (*n* = 400)

Potential associations between the answers given to questions about hearing and vision care and the respondents’ job role, their length of time working in care homes, the type of care home they worked in and also the size of the home were examined by applying the Chi Square test. Table 3 shows which of these care home/staff factors were significantly related to the responses to questions about hearing and vision care. Fourteen of the 31 questions about hearing and vision were significantly related (*p* = 0.000) to one or more of the questions regarding staff characteristics as shown in Table 3. Detailed data, including absolute numbers and relative frequencies, pertaining to all chi-square results are included in Additional file 2.

**Table 3:** Relationships between staff characteristics and question responses (*n* = 400)

Relationships between variables	Job Role	Length of work	Care Home Type	Size of Care Home
*Hearing*				
Use of screening tools	0.000^a^	0.000^a^	0.005	0.099
Annual professional assessment	0.000^a^	0.000^a^	0.002	0.014
Recorded in care plan	0.029	0.378	0.937	0.181
Access to quiet rooms	0.138	0.000^a^	0.387	0.058
Aids checked regularly	0.338	0.000^a^	0.000^a^	0.829
Access to other aids	0.000^a^	0.002	0.003	0.308
Knowledge of different hearing problems	0.349	0.102	0.460	0.176
Knowledge of identifying hearing loss	0.333	0.16	0.544	0.083
Knowledge of identifying hearing loss in the cognitively impaired	0.201	0.152	0.995	0.012
Knowledge of how to clean hearing aids	0.008	0.014	0.480	0.736
Knowledge of how to communicate	0.114	0.067	0.000^a^	0.542
Would like more information	0.078	0.071	0.003	0.244
Family members assist	0.094	0.937	0.577	0.004
Residents willing to use aid	0.003	0.07	0.000^a^	0.004
Can residents take care of own aid	0.000^a^	0.1	0.000^a^	0.554
*Vision*				
Use of screening tools	0.000^a^	0.000^a^	0.153	0.311
Annual professional assessment	0.000^a^	0.000^a^	0.133	0.073
Recorded in care plan	0.214	0.047	0.930	0.655
Environment well lit	0.265	0.010	0.652	0.002
Environment adaptations	0.025	0.021	0.02	0.537
Glasses labelled	0.003	0.000^a^	0.000^a^	0.063
Glasses cleaned regularly	0.1	0.01	0.269	0.825
Access to other aids	0.000^a^	0.000^a^	0.014	0.04
Knowledge of different vision problems	0.000^a^	0.164	0.264	0.123
Knowledge of identifying vision loss	0.414	0.232	0.721	0.311
Knowledge of identifying vision loss in cognitively impaired	0.640	0.650	0.636	0.142
Knowledge of how to clean glasses	0.003	0.013	0.005	0.194
Knowledge of how to communicate	0.088	0.059	0.056	0.042
Would like more information	0.264	0.716	0.265	0.177
Family members assist	0.07	0.254	0.713	0.026
Residents willing to use glasses	0.291	0.694	0.000^a^	0.106

Key: ^a^significant relationships were found

#### Job roles

Four questions about practices related to hearing and four questions about vision practices were significantly associated with job role. As shown in Table 3, whether the care home used screening tools to assess both hearing (*P* = 0.000) and vision difficulties (*P* = 0.000), had access to other hearing (*P* = 0.000) or vision (*P* = 0.000) assistive devices or provided annual hearing (*P* = 0.000) or vision (*P* = 0.000) professional assessments significantly related to job role. A greater number of health care assistants and those classified as ‘other’ stated they were ‘not sure’ regarding whether these three practices for either hearing or vision took place in their employing care home. This was in comparison to care home managers and nurses who tended to state certainty (yes or no responses). However, responses to all other practices identified in the survey; namely, environment adaptations, the checking of assistive aids by staff and reporting of hearing and vision difficulties in care plans, were not found to be significantly related to the job role of the person completing it.

A significant association was found between staff knowledge of different vision problems and their job role (*P* = 0.000). A greater number of care home managers and nurses reported that they had good knowledge of how to identify the different vision problems in their residents in comparison to health care assistants, activities coordinators and those identifying as ‘other’. Furthermore, a greater number of care home managers, compared to other job roles, reported that they believed their residents unable to take care of their own hearing aid (P = 0.000).

Despite these significant results found, job role did not have effect on many of the factors assessed. For instance, all respondents despite job role, responded similarly to views on the environment, including the lighting and accessibility to quiet rooms. Their knowledge was also reported as similar despite job role, including their confidence in cleaning aids, assessing hearing and vision difficulties in residents and confidence in communicating with residents with hearing and vision difficulties.

#### Length of time working in care homes

Significant associations were found between length of work and the three particular practices also affected by job role; namely use of screening tools, professional assessments by ear and eye care experts, and access to other assistive devices. A different level of certainty was again found in the reporting of hearing (*P* = 0.000) and vision (0.000) screening tools, professional hearing (0.000) and vision assessments (*P* = 0.000), and also whether residents have access to other additional vision assistive devices (*P* = 0.000), dependent on length of time the participant reported as working in the care home. Those who had worked in cate homes less than 2 years were most likely to be uncertain about whether these practices were implemented in their care home, whereas those working in the care home for over 10 years were the most certain of the practices being used in their care home.

Significant associations were also found in the reporting of whether hearing aids are checked regularly (*P* = 0.000) and whether the care home has quiet rooms available to residents with hearing difficulties (*P* = 0.000), with greater uncertainty reported by those who had worked in the care home for less than 2 years in comparison to those with greater experience (2–5 years, 6–10 years, 10+ years). The heightened level of uncertainty among those with the least length of service is also evident in the reporting of whether residents’ glasses are labelled (*P* = 0.000).

Whilst some associations were found, knowledge again seemed not to be affected by the respondents’ length of time working in care homes, instead only affecting their knowledge of what practices are available to them and their residents which is evident by the frequency of “not sure” responses. They all also similarly viewed the capabilities and willingness of their residents to wear and take care of their own assistive aids.

#### Type of care home

Care home type was shown to be significantly related to four questions about hearing and two about vision. Respondents from care homes specifically for people with dementia reported that their residents were more likely not to use their prescribed hearing aids (*P* = 0.000) and glasses (*P* = 0.000) in comparison to all other care home types. A greater percentage of respondents from these homes caring specifically for people with dementia also reported that their residents were unable to take care of their own prescribed hearing aids (*P* = 0.000) and were more likely to have their glasses labeled (*P* = 0.000) in comparison to all other care home types.

Respondents from homes noted as caring specifically for those with dementia or as ‘other’ were the least likely to state that they were ‘unsure’ regarding whether residents hearing aids were checked regularly by staff (*P* = 0.000). Staff working in those care homes identifying as ‘other’ were also less likely to be confident in their ability to communicate with residents with hearing difficulties in comparison to respondents from all other care home types (*P* = 0.000).

Care home type however, was not associated with care practices provided to residents, including the use of screening tools, access to other assistive devices, the environment, recording of impairments in care plans, and the accessibility to professional vision assessments. Knowledge again was not associated with care home type, with similar reporting of knowledge of the different conditions, how to assess hearing and vision, how to take care of the different assistive devices and communicate with those with vision difficulties.

#### Size of the care home

Size of care home was not found to be significantly related to any question, and therefore responses were not affected by how many residents resided in the respondents’ care home.

## Discussion

In our cross-sectional study exploring hearing and vision care practices in 74 care homes across England, 400 care staff participated. Our study results showed that whilst some practices known to support residents hearing and vision [4] are commonly adopted by care homes, others were reported as being rarely found to be used. Practices commonly reported to be used include; recording sensory loss in residents’ care plans, annual professional vision tests, regularly checking hearing aids and regularly checking glasses. The majority of respondents also reported having quiet areas and well-lit rooms in their care home. These responses show that some practices suggested by existing guidelines are being adhered to [19]. However, there are certain practices which are known to assist with residents’ ear and eye care that were reportedly rarely used, including use of screening tools for both hearing and vision, hearing assessments by audiologists, and access to other assistive devices. This reveals that interventions aimed at improving practice, including use of screening tools as identified through the scoping review [15, 16], are rarely being implemented in practice. Our analysis also showed that there is more support available to identify and manage residents’ vision than hearing. As the study assessed which practices found to affect ear and eye care are adhered to in care homes, participants responses revealed that there are more vision practices being adhered to in practice when compared to adherence of hearing practices. However, both hearing and vision of care home residents can be neglected, with some screening tools, additional assistive devices and professional assessments not commonly used/provided.

Participants also stated that their knowledge was lacking in certain areas. Firstly, the majority were not confident in identifying and managing both hearing and vision impairments in their residents, which could contribute to the poor care of residents hearing and vision difficulties in care home residents [21]. This further highlights, that despite interventions aiming to improve knowledge in staff being reported as effective [11–13], these have not been implemented effectively into practice across the country. In addition, audiology services were commonly reported as not being readily available to residents, suggesting this may be a further reason for the poor identification of hearing impairments in residents [6]. Whilst optometry services are commissioned to provide annual assessments in care homes in England, as confirmed in this study, audiology services are not commissioned or funded to conduct assessments in care homes and has evidently had an effect on how often these are carried out [24]. The poor adoption of some prescribed aids (i.e. hearing loop systems), and the reported limited access to other assistive devices to compensate for hearing or sight loss also suggests that residents may not be using devices that can improve or compensate for their sensory impairment. This can be another reason for poorly managed sensory impairments in residents [25].

Hearing aids and glasses were checked regularly by staff but the majority stated that they are not entirely confident in how these aids should be cleaned and maintained, and in particular hearing aids. This suggests that guidelines and interventions aimed at improving aid management need to be formally implemented, making this mandatory and increasing support for their acceptance in practice [6, 19]. So, whilst checked, these aids may not be functioning as they should be, resulting in residents still living with the effects of sensory impairments. This suggests that staff might need enhanced training to provide a sufficiently skilled service. The vast majority of respondents also stated they would like more information on how to identify and manage both hearing and vision loss, highlighting a need amongst all staff for further training in more than just aid management. This suggests that care home staff and researchers should prioritise providing training for care home staff in ear and eye care; this could also include developing the skills and knowledge of a particular staff member within each care home to create hearing and vision care champions within the team who can address ear and eye concerns effectively and efficiently.

The high level of uncertainty amongst health care assistants and those identifying as ‘other’ on the practices used in care homes, including whether screening tools, other assistive devices and professional assessments are used in their care home for both hearing and vision, also suggests that there may be a poor level of shared communication about these practices. Health care assistants, including the senior health care assistants who identify within the group ‘other’, are often the first point of contact for residents and so need to be made aware as a priority. A champion amongst the health care assistants in ear and eye care may aid to reduce this lack of shared communication within the care home, and increase knowledge amongst these groups of the practices available within and outside of the home that could be useful when providing personal cares. However, no difference in knowledge of hearing and vision difficulties and how to manage them was found between job role, emphasising that hearing and vision loss knowledge is the same despite job role.

Homes catering exclusively for people with dementia reported the lowest willingness among residents to use and also care for their prescribed aids, which reveals that cognition may play a significant role in residents’ ability to use and maintain ownership of their aids, suggesting that staff in homes for people with dementia may need to have heightened awareness and training. Respondents from dementia only homes also report a lesser likelihood of audiologists providing annual assessments in comparison to general residential or nursing homes. As cognitive impairment is known to be a further barrier to effective identification and management [26], these results seem to suggest that staff and other professionals need to be trained specifically in dementia care [27]. This may include providing training on how to recognise sensory impairments in those living with dementia and knowing the difference between what might be a sensory impairment and what is dementia. Also, how to screen those with memory difficulties for hearing and vision difficulties and how to encourage assistive aid use.

The level of knowledge of hearing and vision care among all respondents was similar irrespective of their job role, length of time working in the home, care home type or size of their home. Therefore, whilst knowledge of staff was found to be poor for identification and management of both hearing and vision loss, this was the same regardless of participant or care home characteristics. The results show that it is mainly the respondents’ knowledge of whether certain practices are adopted in their care home that differs dependent on the different demographic factors. This suggests there is a universal need for training and awareness of the importance of shared communication regarding practices available within the care home to support with residents’ ear and eye care.

The study results suggest that to improve hearing and vision care practices a focus on improving certain aspects is required. These include; having a range of assistive devices available in care homes such as hearing loop systems and magnifying glasses, having screening tools that all staff can use in their daily practice to identify those at risk, building stronger links between care homes and external professionals, and training care staff to increase their knowledge of how to both identify and manage hearing and vision impairments effectively. With certain responses affected by the individual profiles of the respondents, it also highlights a lack of standardised procedure and shared knowledge across care homes and their staff. Therefore, improved communication and best practices for hearing and vision care need to be implemented to ensure effective hearing and vision care is provided by all care staff. This could be achieved with a change in policy and guidelines that inform managers of the best practices [4], and the importance of informing their staff of the practices available to care for residents hearing and vision within their home.

### Limitations

Our study has several limitations to consider, including:

The survey technique used, that of predetermined questions, limits the participants’ capacity to present details of their knowledge and understanding. This includes other barriers that they may experience but were not included within the survey, such as lack of time or human resources, and information on any training they may have received. Staffing ratio was also not assessed which could have affected the findings, with those with higher staffing ratio possibly providing a different level of care to those with lower staffing ratios. It was not possible to determine the numbers who had received the survey and not completed it as methods of distribution within each region was dependent on the clinical networks method of distribution and within each home was dependent on the manager, and so non-respondent data could not be calculated. Furthermore, certain postcodes were not completed correctly by some respondents and led to some care homes being unidentified. This meant that CQC ratings could not be obtained for all care homes and discrepancies of care home type unable to be rectified by internet searching for these few respondents. In addition, only 74 care homes took part, all of which were recruited via convenience sampling which may not be a true representation of all care homes across England.

Nevertheless, this study obtained national data from a variety of care homes, which provides a good scope of the variety of the facilitators to good quality ear and eye care and the barriers to this from the perspectives of staff working in different settings. In addition, by using questions that attained to themes derived from the scoping review provided a framework for the survey [4], and this was validated by an advisory group comprised of intended respondents.

## Conclusion

Our study results indicate that whilst some services are available in most care homes for ear and eye care across England, others are not as frequently implemented or that staff are unsure of whether such services are available or not. This highlights the underuse of the certain services and implies patchy implementation of the practices which could significantly benefit residents hearing and vision capabilities and improve mental and physical health. Consequently, this infers that best practice for ear and eye care in care home residents can be improved, and this evidence identifies specifically which practices should be targeted, including screening tools, annual hearing tests conducted by external services and having access to various devices. In addition, staff knowledge shows room for improvement, with the majority of staff wanting more information on how to care for both hearing and vision in their residents. This study confirms the need for better support for care home staff so that they can provide optimum ear and eye care including services provided by others external to the home. It also identifies a need for further research to explore how quality services can be both provided and sustained over time. Due to the limited evidence, the implementation of mentioned practices should be evaluated by research on the effectiveness and benefit for residents with regard to hearing and vision. This could also focus on the improvement of mental and physical health and residents’ quality of life.

## Supplementary Information

**Additional file 1.** Survey. This is the hard copy version of the survey that was distributed to care home staff.

**Additional file 2.** Absolute Numbers and Relative Frequencies found in Chi Square Analyses. This file contains additional statistical data from the analyses conducted.

## Data Availability

The datasets used and/or analysed during the current study are available from the corresponding author on reasonable request.

## Abbreviations

CQC: Care Quality Commission
ENRICH: Enabling Research in Care Homes Network
NHS: National Health Service
