# Supplementary material for: Hearing and vision care provided to older people residing in care homes: a cross-sectional survey of care home staff
Source: BMC Geriatr. 2021 Jan 8;21:32. doi: 10.1186/s12877-020-01959-0 (PMC7791835; doi:10.1186/s12877-020-01959-0)
Supplement: Supplementary file 1 — Additional file 1. Survey. This is the hard copy version of the survey that was distributed to care home staff. [file 12877_2020_1959_MOESM1_ESM.pdf]

Caring for Hearing and Vision Loss in Care Homes (hard copy)

Page 1: Privacy notice

The aim of this survey is to gather your feedback on the care of individuals with hearing and vision loss in your care home. It should take approximately five minutes to complete. The survey consists of 33 multiple choice questions about identifying hearing and vision loss in residents, and how these sensory losses are cared for once identified in your care home. Please select only one option for each of the questions.

There are three sections to this survey. The first will ask an additional six questions about your specific care home, but please note this will **NOT** include any requests for personal information. These questions ask about your role and the type of care home you work in. It will also ask for the postcode of your care home, this will allow us to acknowledge your care home's participation in this research which will be nationally recorded and valued.

The feedback you provide will be anonymous as your name and contact details will not be collected. All data gathered will be stored in a password protected electronic format, with access only granted to the researcher (Wendy Andrusjak) and three academic supervisors. The results of this survey will be used for scholarly purposes only, which may include publication in medical journals.

If you have any queries about taking part in this survey please contact Wendy Andrusjak at W.andrusjak@bradford.ac.uk.

To progress with the survey please ensure you agree to take part by consenting below.

1. Ticking the "I agree" box below indicates that you have read the above information, you voluntarily agree to take part, and that you are 18 years or over. If you do not wish to take

part in the study, please decline participation by ticking the "I disagree" box.

- I Agree
- I Disagree

Page 2: Details

2. How would you describe your job role? ✱ *Required*

- ◯ Care Home Manager
- ◯ Qualified Nurse
- ◯ Health Care Assistant
- ◯ Activities Coordinator
- ◯ Other

3. How long have you worked in care homes? ✱ *Required*

- ◯ Less than 2 years
- ◯ 2-5 years
- ◯ 6-10 years
- ◯ 10+ years

4. What type of care home do you work in? ✱ *Required*

- ☐ Nursing Home
- ☐ Residential Care Home
- ☐ A Dementia Only Care Home
- ☐ Other

5. How many residents currently reside in your care home? ✱ *Required*

- ◯ 0-30

- 31-60
- 61+

6. The majority of the care home residents are over the age of 65 years old ✱ *Required*

- Yes
- No

7. What is the postcode of your care home? *Optional*

Page 3: Hearing Loss

The following statements are related to hearing loss in care homes, please select the most relevant response to you.

8. I know how to tell the difference between the various hearing problems (i.e. age-related, tinnitus, and ear wax build up) ✱ *Required*

- Strongly Agree
- Agree
- Disagree
- Strongly Disagree

9. I am confident in assessing whether a resident has hearing difficulties ✱ *Required*

- Strongly Agree
- Agree
- Disagree
- Strongly Disagree

10. I am confident in assessing whether a resident with cognitive difficulties (i.e. dementia) has hearing difficulties ✱ *Required*

- Strongly Agree
- Agree
- Disagree
- Strongly Disagree

11. The care home use screening tools to identify hearing loss ✱ *Required*

- Yes
- No
- Not Sure

12. Residents hearing difficulties are recorded in their care plan ✱ *Required*

- Yes
- Sometimes
- No
- Not Sure

13. The care home has dedicated public quiet areas ✱ *Required*

- Yes
- No
- Not Sure

14. I am confident in cleaning hearing aids ✱ *Required*

- Strongly Agree
- Agree
- Disagree
- Strongly Disagree

15. Hearing aids are used by some of our residents ✱ *Required*

- Yes
- No
- Not Sure

16. Residents' are willing to use their hearing aids provided ✱ *Required*

- All
- Some
- Very few
- None

17. Hearing aids are checked regularly by care staff to see if they are working correctly ✱ *Required*

- Yes
- No
- Not Sure

18. The majority of residents are able to take care of their own hearing aids ✱ *Required*

- Strongly Agree
- Agree
- Disagree
- Strongly Disagree

19. The residents have access to other hearing aids/devices such as headphones and hearing loop systems ✱ *Required*

- Yes
- Only when the residents' bring their own
- No
- Not Sure

20. Family members recognise and assist with their relatives hearing problems ✱ *Required*

- Yes
- Sometimes
- No

21. All residents have annual hearing check-ups by professional audiologists ✱ *Required*

- Yes
- No
- Not Sure

22. I am confident in communicating with residents with hearing difficulties ✱ *Required*

- I am confident
- I am confident but would like more information
- I am not confident but I know the basics
- I am not confident at all

23. I would like more information on how to better identify and manage hearing loss ✱
Required

- Strongly Agree
- Agree
- Disagree
- Strongly Disagree

Page 4: Vision Loss

The following statements are related to vision loss in care homes, please select the most relevant response to you.

24. I know how to tell the difference between the various vision problems (i.e. cataracts, macular degeneration, and glaucoma) ✱ *Required*

- Strongly Agree
- Agree
- Disagree
- Strongly Disagree

25. I am confident in assessing whether a resident is poorly sighted ✱ *Required*

- Strongly Agree
- Agree
- Disagree
- Strongly Disagree

26. I am confident in assessing whether a resident with cognitive difficulties (i.e. dementia) is poorly sighted ✱ *Required*

- Strongly Agree
- Agree
- Disagree
- Strongly Disagree

27. The care home use screening tools to identify vision loss ✱ *Required*

- Yes
- No
- Not Sure

28. Residents poor vision is recorded in their care plan ✱ *Required*

- Yes
- Sometimes
- No
- Not Sure

29. The care home is well lit to cater for residents with poor vision ✱ *Required*

- Yes
- No
- Not Sure

30. Adaptations to the environment, such as extra-large font corridor signs or newsletters, are used around the care home ✱ *Required*

- Yes
- No
- Not Sure

31. I am confident in cleaning vision aids such as residents glasses ✱ *Required*

- Strongly Agree
- Agree
- Disagree
- Strongly Disagree

32. Glasses are used by some of our residents ✱ *Required*

- Yes
- No
- Not Sure

33. Residents' are willing to use their glasses provided ✱ *Required*

- All
- Some
- Very Few
- None

34. Residents glasses are cleaned regularly by care staff ✱ *Required*

- Yes
- No
- Not Sure

35. Residents glasses are labelled with their names ✱ *Required*

- Yes
- Some
- No
- Not Sure

36. Residents have access to other assisstive devices such as large font books or magnifying glasses ✱ *Required*

- Yes
- Only when the residents' bring their own
- No
- Not Sure

37. Family members recognise and assist with their relatives vision problems ✱ *Required*

- Strongly Agree
- Agree
- Disagree
- Strongly Disagree

38. All residents have annual vision check-ups by professional optometrists ✱ *Required*

- Yes
- No
- Not Sure

39. I am confident in communicating with residents with poor vision ✱ *Required*

- I am confident
- I am confident but would like more information
- I am not confident but I know the basics
- I am not confident at all

40. I would like more information on how to better identify and manage vision loss ✱ *Required*

- Strongly Agree
- Agree
- Disagree
- Strongly Disagree

41. Do you have any other services you/your care home provide to assist those with hearing and vision difficulties? *Optional*

Page 5: Thank you for completing this survey
