# Supplementary material for: Hearing and vision care provided to older people residing in care homes: a cross-sectional survey of care home staff
Source: BMC Geriatr. 2021 Jan 8;21:32. doi: 10.1186/s12877-020-01959-0 (PMC7791835; doi:10.1186/s12877-020-01959-0)
Supplement: Supplementary file 2 — Additional file 2. Absolute Numbers and Relative Frequencies found in Chi Square Analyses. This file contains additional statistical data from the analyses conducted. [file 12877_2020_1959_MOESM2_ESM.docx]

**Additional file 2**

**Chi Square output**

* Significant result

**Job role**

I know how to tell the difference between the various hearing problems

| Knowledge of hearing problems (%/n) | Care Home Manager | Registered Nurse | Health Care Assistant | Activities Coordinator | Other |
| --- | --- | --- | --- | --- | --- |
| Strongly Agree | 21.5 (14) | 21.4 (12) | 16.2 (34) | 7.1 (1) | 16.4 (9) |
| Agree | 50.8 (33) | 66.1 (37) | 56.2 (118) | 50 (7) | 52.7 (29) |
| Disagree | 27.7 (18) | 10.7 (6) | 25.7 (54) | 35.7 (5) | 27.3 (15) |
| Strongly Disagree | 0 (0) | 1.8 (1) | 1.9 (4) | 7.1 (1) | 3.6 (2) |

I am confident in assessing whether a resident has hearing difficulties

| Identifying hearing problems (%/n) | Care Home Manager | Registered Nurse | Health Care Assistant | Activities Coordinator | Other |
| --- | --- | --- | --- | --- | --- |
| Strongly Agree | 33.8 (22) | 26.8 (15) | 22.4 (47) | 28.6 (4) | 20 (11) |
| Agree | 50.8 (33) | 64.3 (36) | 63.3 (133) | 50 (7) | 56.4 (31) |
| Disagree | 15.4 (10) | 8.9 (5) | 11.4 (24) | 21.4 (3) | 20 (11) |
| Strongly Disagree | 0 (0) | 0 (0) | 2.9 (6) | 0 (0) | 3.6 (2) |

I am confident in assessing whether a resident with cognitive difficulties has hearing difficulties

| Identifying hearing problems in cognitively impaired (%/n) | Care Home Manager | Registered Nurse | Health Care Assistant | Activities Coordinator | Other |
| --- | --- | --- | --- | --- | --- |
| Strongly Agree | 29.2 (19) | 26.8 (15) | 17.6 (37) | 14.3 (2) | 23.6 (13) |
| Agree | 44.6 (29) | 58.9 (33) | 60 (126) | 57.1 (8) | 43.6 (24) |
| Disagree | 26.2 (17) | 12.5 (7) | 19.5 (41) | 28.6 (4) | 29.1 (16) |
| Strongly Disagree | 0 (0) | 1.8 (1) | 2.9 (6) | 0 (0) | 3.6 (2) |

The care home use screening tools to identify hearing loss *

| Screening tools (%/n) | Care Home Manager | Registered Nurse | Health Care Assistant | Activities Coordinator | Other |
| --- | --- | --- | --- | --- | --- |
| Yes | 18.5 (12) | 28.6 (16) | 13.8 (29) | 21.4 (3) | 7.3 (4) |
| No | 75.4 (49) | 62.5 (35) | 36.2 (76) | 28.6 (4) | 36.4 (20) |
| Not Sure | 6.2 (4) | 8.9 (5) | 50 (105) | 50 (7) | 56.4 (31) |

Residents hearing difficulties are recorded in their care plan

| Care plan (%/n) | Care Home Manager | Registered Nurse | Health Care Assistant | Activities Coordinator | Other |
| --- | --- | --- | --- | --- | --- |
| Yes | 95.4 (62) | 83.9 (47) | 91.4 (192) | 100 (14) | 90.9 (50) |
| No | 0 (0) | 0 (0) | 0.5 (1) | 0 (0) | 0 (0) |
| Not Sure | 0 (0) | 0 (0) | 1.9 (4) | 0 (0) | 7.3 (4) |
|  | 4.6 (3) | 16.1 (9) | 6.2 (13) | 0 (0) | 1.8 (1) |

The care home has dedicated public quiet areas

| Environment (%/n) | Care Home Manager | Registered Nurse | Health Care Assistant | Activities Coordinator | Other |
| --- | --- | --- | --- | --- | --- |
| Yes | 87.7 (57) | 78.6 (44) | 75.2 (158) | 92.9 (13) | 83.6 (46) |
| No | 12.3 (8) | 17.9 (10) | 15.7 (33) | 0 (0) | 10.9 (6) |
| Not Sure | 0 (0) | 3.6 (2) | 7.1 (1) | 7.1 (1) | 5.5 (3) |

I am confident in cleaning hearing aids

| Knowledge cleaning aids (%/n) | Care Home Manager | Registered Nurse | Health Care Assistant | Activities Coordinator | Other |
| --- | --- | --- | --- | --- | --- |
| Strongly Agree | 30.8 (20) | 28.6 (16) | 22.4 (47) | 21.4 (3) | 16.4 (9) |
| Agree | 58.5 (38) | 64.3 (36) | 52.4 (110) | 42.9 (6) | 47.3 (26) |
| Disagree | 7.7 (5) | 5.4 (3) | 21.4 (45) | 21.4 (3) | 32.7 (18) |
| Strongly Disagree | 3.1 (2) | 1.8 (1) | 2.9 (6) | 14.3 (2) | 3.6 (2) |

Residents are willing to use their hearing aids provided

| Residents Willing (%/n) | Care Home Manager | Registered Nurse | Health Care Assistant | Activities Coordinator | Other |
| --- | --- | --- | --- | --- | --- |
| All | 9.2 (6) | 12.5 (7) | 32.4 (68) | 42.9 (6) | 29.1 (16) |
| Some | 73.8 (48) | 78.6 (44) | 60 (126) | 57.1 (8) | 67.3 (37) |
| Very few | 12.3 (8) | 7.1 (4) | 6.7 (14) | 0 (0) | 3.6 (2) |
| None | 4.6 (3) | 1.8 (1) | 1 (2) | 0 (0) | 0 (0) |

Hearing aids are checked regularly by care staff to see if they are working correctly

| Aids checked regularly (%/n) | Care Home Manager | Registered Nurse | Health Care Assistant | Activities Coordinator | Other |
| --- | --- | --- | --- | --- | --- |
| Yes | 84.6 (55) | 92.9 (52) | 82.9 (174) | 78.6 (11) | 80 (44) |
| No | 7.7 (5) | 1.8 (1) | 5.7 (12) | 0 (0) | 3.6 (2) |
| Not sure | 7.7 (5) | 5.4 (3) | 11.4 (24) | 21.4 (3) | 16.4 (9) |

| Residents care for own aid (%/n) | Care Home Manager | Registered Nurse | Health Care Assistant | Activities Coordinator | Other |
| --- | --- | --- | --- | --- | --- |
| Strongly Agree | 0 (0) | 0 (0) | 4.3 (9) | 0 (0) | 0 (0) |
| Agree | 7.7 (5) | 17.9 (10) | 21.9 (46) | 7.1 (1) | 21.8 (12) |
| Disagree | 52.3 (34) | 60.7 (34) | 60.5 (127) | 92.9 (13) | 74.5 (41) |
| Strongly disagree | 40 (26) | 21.4 (12) | 13.3 (28) | 0 (0) | 3.6 (2) |

The majority of residents are able to take care of their own hearing aids *

| Access to other aids (%/n) | Care Home Manager | Registered Nurse | Health Care Assistant | Activities Coordinator | Other |
| --- | --- | --- | --- | --- | --- |
| Yes | 20 (13) | 12.5 (7) | 11.9 (25) | 21.4 (3) | 21.8 (12) |
| No | 35.4 (23) | 37.5 (21) | 28.1 (59) | 21.4 (3) | 23.6 (13) |
| Not Sure | 0 (0) | 1.8 (1) | 25.2 (53) | 42.9 (6) | 27.3 (15) |
| Only when they bring their own | 44.6 (29) | 48.2 (27) | 34.8 (73) | 14.3 (2) | 27.3 (15) |

The residents have access to other hearing aids/devices such as headphones and hearing loop systems *

Family members recognise and assist with their relatives hearing problems

| Family members assist (%/n) | Care Home Manager | Registered Nurse | Health Care Assistant | Activities Coordinator | Other |
| --- | --- | --- | --- | --- | --- |
| Yes | 29.2 (19) | 55.4 (31) | 40.5 (85) | 42.9 (6) | 41.8 (23) |
| No | 3.1 (2) | 3.6 (2) | 5.7 (12) | 0 (0) | 0 (0) |
| Sometimes | 67.7 (44) | 41.1 (23) | 53.8 (113) | 57.1 (8) | 58.2 (32) |

All residents have annual hearing check-ups by professional audiologists *

| Professional assessment (%/n) | Care Home Manager | Registered Nurse | Health Care Assistant | Activities Coordinator | Other |
| --- | --- | --- | --- | --- | --- |
| Yes | 33.8 (22) | 46.4 (26) | 52.4 (110) | 42.9 (6) | 41.8 (23) |
| No | 61.5 (40) | 46.4 (26) | 11 (23) | 14.3 (2) | 16.4 (9) |
| Not Sure | 4.6 (3) | 7.1 (4) | 36.7 (77) | 42.9 (6) | 41.8 (23) |

I am confident in communicating with residents with hearing difficulties

| Knowledge communicating (%/n) | Care Home Manager | Registered Nurse | Health Care Assistant | Activities Coordinator | Other |
| --- | --- | --- | --- | --- | --- |
| I am confident | 49.2 (2.8) | 64.3 (36) | 67.1 (141) | 78.6 (11) | 61.8 (34) |
| I am confident but would like more information | 44.6 (29) | 33.9 (19) | 26.2 (55) | 21.4 (3) | 25.5 (14) |
| I am not confident, but I know the basics | 6.2 (4) | 0 (0) | 12 (1) | 0 (0) | 6 (3) |
| I am not confident at all | 0 (0) | 1.8 (1) | 1 (2) | 0 (0) | 1.8 (1) |

I would like more information on how to better identify and manage hearing loss

| Want more information (%/n) | Care Home Manager | Registered Nurse | Health Care Assistant | Activities Coordinator | Other |
| --- | --- | --- | --- | --- | --- |
| Strongly Agree | 35.4 (23) | 23.2 (13) | 26.7 (56) | 50 (7) | 16.4 (9) |
| Agree | 61.5 (40) | 69.6 (39) | 61.4 (129) | 35.7 (5) | 67.3 (37) |
| Disagree | 1 (0.5) | 5.4 (3) | 11 (23) | 14.3 (2) | 16.4 (9) |
| Strongly Disagree | 1.5 (1) | 1.8 (1) | 1 (2) | 0 (0) | 0 (0) |

Vision

I know how to tell the difference between the various vision problems (i.e. cataracts, macular degeneration, and glaucoma) *

| Knowledge of vision problems (%/n) | Care Home Manager | Registered Nurse | Health Care Assistant | Activities Coordinator | Other |
| --- | --- | --- | --- | --- | --- |
| Strongly Agree | 18.5 (12) | 12.5 (7) | 5.7 (12) | 7.1 (1) | 1.8 (1) |
| Agree | 50.8 (33) | 60.7 (34) | 35.7 (75) | 35.7 (5) | 45.5 (25) |
| Disagree | 27.7 (18) | 21.4 (12) | 50 (105) | 50 (7) | 47.3 (26) |
| Strongly Disagree | 3.1 (2) | 5.4 (3) | 8.6 (18) | 7.1 (1) | 5.5 (3) |

I am confident in assessing whether a resident is poorly sighted

| Identifying vision problems (%/n) | Care Home Manager | Registered Nurse | Health Care Assistant | Activities Coordinator | Other |
| --- | --- | --- | --- | --- | --- |
| Strongly Agree | 20 (13) | 16.1 (9) | 11 (23) | 14.3 (2) | 16.4 (9) |
| Agree | 53.8 (35) | 60.7 (34) | 63.3 (133) | 71.4 (10) | 45.5 (25) |
| Disagree | 26.2 (17) | 21.4 (12) | 23.3 (49) | 14.3 (2) | 36.4 (20) |
| Strongly Disagree | 0 (0) | 1.8 (1) | 2.4 (5) | 0 (0) | 1.8 (1) |

I am confident in assessing whether a resident with cognitive difficulties is poorly sighted

| Identifying vision problems in cognitively impaired (%/n) | Care Home Manager | Registered Nurse | Health Care Assistant | Activities Coordinator | Other |
| --- | --- | --- | --- | --- | --- |
| Strongly Agree | 20 (13) | 17.9 (10) | 12.4 (26) | 14.3 (2) | 12.7 (7) |
| Agree | 49.2 (32) | 55.4 (31) | 53.3 (112) | 57.1 (8) | 43.6 (24) |
| Disagree | 30.8 (20) | 25 (14) | 30 (63) | 286 (4) | 40 (22) |
| Strongly Disagree | 0 (0) | 1.8 (1) | 4.3 (9) | 0 (0) | 3.6 (2) |

The care home use screening tools to identify vision loss *

| Screening tools (%/n) | Care Home Manager | Registered Nurse | Health Care Assistant | Activities Coordinator | Other |
| --- | --- | --- | --- | --- | --- |
| Yes | 33.8 (22) | 30.4 (17) | 21.9 (46) | 14.3 (2) | 14.5 (8) |
| No | 60 (39) | 60.7 (34) | 37.1 (78) | 21.4 (3) | 38.2 (21) |
| Not Sure | 6.2 (4) | 8.9 (5) | 41 (86) | 64.3 (9) | 47.3 (26) |

Residents poor vision is recorded in their care plan

| Care plan (%/n) | Care Home Manager | Registered Nurse | Health Care Assistant | Activities Coordinator | Other |
| --- | --- | --- | --- | --- | --- |
| Yes | 93.8 (61) | 98.2 (55) | 92.4 (194) | 92.9 (13) | 92.7 (51) |
| Sometimes | 4.6 (3) | 1.8 (1) | 4.8 (10) | 0 (0) | 0 (0) |
| No | 1.5 (1) | 0 (0) | 0.5 (1) | 0 (0) | 0 (0) |
| Not sure | 0 (0) | 0 (0) | 2.4 (5) | 7.1 (1) | 7.3 (4) |

The care home is well lit to cater for residents with poor vision

| Environment well lit (%/n) | Care Home Manager | Registered Nurse | Health Care Assistant | Activities Coordinator | Other |
| --- | --- | --- | --- | --- | --- |
| Yes | 90.8 (59) | 91.1 (51) | 84.3 (177) | 92.9 (13) | 96.4 (53) |
| No | 6.2 (4) | 3.6 (2) | 6.2 (13) | 7.1 (1) | 1.8 (1) |
| Not sure | 3.1 (2) | 5.4 (3) | 9.5 (20) | 0 (0) | 1.8 (1) |

Adaptations to the environment, such as extra-large font corridor signs or newsletter, are used around the care home

| Environment adaptations (%/n) | Care Home Manager | Registered Nurse | Health Care Assistant | Activities Coordinator | Other |
| --- | --- | --- | --- | --- | --- |
| Yes | 76.9 (50) | 69.6 (39) | 62.4 (131) | 71.4 (10) | 74.5 (41) |
| No | 23.1 (15) | 25 (14) | 21.9 (46) | 14.3 (2) | 18.2 (10) |
| Not sure | 0 (0) | 5.4 (3) | 15.7 (33) | 14.3 (2) | 7.3 (4) |

I am confident in cleaning vision aids such as residents glasses

| Knowledge cleaning glasses (%/n) | Care Home Manager | Registered Nurse | Health Care Assistant | Activities Coordinator | Other |
| --- | --- | --- | --- | --- | --- |
| Strongly Agree | 78.5 (51) | 55.4 (31) | 53.3 (112) | 64.3 (9) | 49.1 (27) |
| Agree | 21.5 (14) | 42.9 (24) | 43.8 (92) | 35.7 | 41.8 (23) |
| Disagree | 0 (0) | 0 (0) | 1.9 (4) | 0 (0) | 9.1 (5) |
| Strongly Disagree | 0 (0) | 1.8 (1) | 1 (2) | 0 (0) | 0 (0) |

Residents are willing to use their glasses provided

| Residents Willing (%/n) | Care Home Manager | Registered Nurse | Health Care Assistant | Activities Coordinator | Other |
| --- | --- | --- | --- | --- | --- |
| All | 16.9 (11) | 30.4 (17) | 31.9 (67) | 28.6 (4) | 29.1 (16) |
| Some | 83.1 (54) | 62.5 (35) | 63.8 (35) | 71.4 (10) | 67.3 (37) |
| Very few | 0 (0) | 7.1 (4) | 3.8 (8) | 0 (0) | 1.8 (1) |
| None | 0 (0) | 0 (0) | 0.5 (1) | 0 (0) | 0 (0) |

Residents glasses are cleaned regularly by care staff

| Glasses cleaned regularly (%/n) | Care Home Manager | Registered Nurse | Health Care Assistant | Activities Coordinator | Other |
| --- | --- | --- | --- | --- | --- |
| Yes | 95.4 (62) | 96.4 (54) | 91.4 (192) | 85.7 (12) | 85.5 (47) |
| No | 3.1 (2) | 1.8 (1) | 5.7 (12) | 0 (0) | 5.5 (3) |
| Not sure | 1.5 (1) | 1.8 (1) | 2.9 (6) | 14.3 (2) | 9.1 (5) |

Residents glasses are labelled with their names

| Glasses labelled (%/n) | Care Home Manager | Registered Nurse | Health Care Assistant | Activities Coordinator | Other |
| --- | --- | --- | --- | --- | --- |
| Yes | 46.2 (30) | 58.9 (33) | 51 (107) | 50 (7) | 32.7 (18) |
| Some | 53.8 (35) | 35.7 (20) | 35.7 (75) | 35.7 (5) | 45.5 (25) |
| No | 0 (0) | 3.6 (2) | 9.5 (20) | 0 (0) | 12.7 (7) |
| Not sure | 0 (0) | 1.8 (1) | 3.8 (8) | 14.3 (2) | 9.1 (5) |

Residents have access to other assistive devices such as large font books or magnifying glasses *

| Access to other aids (%) | Care Home Manager | Registered Nurse | Health Care Assistant | Activities Coordinator | Other |
| --- | --- | --- | --- | --- | --- |
| Yes | 66.2 (43) | 39.3 (22) | 38.1 (80) | 50 (7) | 50.9 (28) |
| No | 3.1 (2) | 14.3 (8) | 9 (19) | 14.3 (2) | 3.6 (2) |
| Not Sure | 0 (0) | 5.4 (3) | 18.6 (39) | 0 (0) | 21.8 (12) |
| Only when they bring their own | 30.8 (20) | 41.1 (23) | 34.3 (72) | 35.7 (5) | 23.6 (13) |

Family members recognise and assist with their relatives vision problems

| Family members assist (%/n) | Care Home Manager | Registered Nurse | Health Care Assistant | Activities Coordinator | Other |
| --- | --- | --- | --- | --- | --- |
| Strongly Agree | 18.5 (12) | 10.7 (6) | 16.7 (35) | 21.4 (3) | 16.4 (9) |
| Agree | 60 (39) | 85.7 (48) | 73.8 (155) | 71.4 (10) | 76.4 (42) |
| Disagree | 21.5 (14) | 3.6 (2) | 8.1 (17) | 7.1 (1) | 97.3 (4) |
| Strongly Disagree | 0 (0) | 0 (0) | 1.4 (3) | 0 (0) | 0 (0) |

All residents have annual vision check-ups by professional optometrists *

| Professional assessment (%) | Care Home Manager | Registered Nurse | Health Care Assistant | Activities Coordinator | Other |
| --- | --- | --- | --- | --- | --- |
| Yes | 93.8 (61) | 89.3 (50) | 80 (168) | 100 (14) | 87.3 (48) |
| No | 3.1 (2) | 8.9 (5) | 1.4 (3) | 0 (0) | 1.8 (1) |
| Not Sure | 3.1 (2) | 1.8 (1) | 18.6 (39) | 0 (0) | 10.9 (6) |

I am confident in communicating with residents with poor vision

| Knowledge communicating (%/n) | Care Home Manager | Registered Nurse | Health Care Assistant | Activities Coordinator | Other |
| --- | --- | --- | --- | --- | --- |
| I am confident | 56.9 (37) | 67.9 (38) | 74.8 (157) | 78.6 (11) | 70.9 (39) |
| I am confident but would like more information | 41.5 (27) | 30.4 (17) | 21.4 (45) | 21.4 (3) | 20 (11) |
| I am not confident, but I know the basics | 61.5 (1) | 1.8 (1) | 3.3 (7) | 0 (0) | 9.1 (5) |
| I am not confident at all | 0 (0) | 0 (0) | 0.5 (1) | 0 (0) | 0 (0) |

I would like more information on how to better identify and manage vision loss

| Want more information (%/n) | Care Home Manager | Registered Nurse | Health Care Assistant | Activities Coordinator | Other |
| --- | --- | --- | --- | --- | --- |
| Strongly Agree | 36.9 (24) | 19.6 (11) | 25.2 (53) | 14.3 (2) | 21.8 (12) |
| Agree | 55.4 (36) | 71.4 (40) | 57.1 (120) | 71.4 (10) | 60 (33) |
| Disagree | 7.7 (5) | 7.1 (4) | 15.7 (33) | 14.3 (2) | 18.2 (10) |
| Strongly Disagree | 0 (0) | 1.8 (1) | 1.9 (4) | 0 (0) | 0 (0) |

**Length of Work**

Hearing

I know how to tell the difference between the various hearing problems

| Knowledge of hearing problems (%/n) | Less than 2 years | 2-5 years | 6-10 years | 10 + years |
| --- | --- | --- | --- | --- |
| Strongly Agree | 16.7 (13) | 29.5 (36) | 25.8 (16) | 24.6 (34) |
| Agree | 59 (46) | 50 (61) | 62.9 (39) | 56.5 (78) |
| Disagree | 28.2 (22) | 27 (33) | 21 (13) | 21.7 (30) |
| Strongly Disagree | 3.8 (3) | 2.5 (3) | 0 (0) | 1.4 (2) |

I am confident in assessing whether a resident has hearing difficulties

| Identifying hearing problems (%/n) | Less than 2 years | 2-5 years | 6-10 years | 10 + years |
| --- | --- | --- | --- | --- |
| Strongly Agree | 7.7 (6) | 21.3 (26) | 14.5 (9) | 21 (29) |
| Agree | 60.3 (47) | 55.7 (68) | 69.4 (43) | 59.4 (82) |
| Disagree | 19.2 (15) | 12.3 (15) | 4.8 (3) | 14.5 (20) |
| Strongly Disagree | 5.1 (4) | 1.6 (2) | 1.6 (1) | 0.7 (1) |

I am confident in assessing whether a resident with cognitive difficulties has hearing difficulties

| Identifying hearing problems in cognitively impaired (%/n) | Less than 2 years | 2-5 years | 6-10 years | 10 + years |
| --- | --- | --- | --- | --- |
| Strongly Agree | 10.3 (8) | 25.4 (31) | 25.8 (16) | 22.5 (31) |
| Agree | 60.3 (47) | 50.8 (62) | 62.9 (39) | 52.2 (72) |
| Disagree | 26.9 (21) | 12.3 (15) | 9.7 (6) | 23.2 (32) |
| Strongly Disagree | 2.6 (2) | 2.5 (3) | 1.6 (1) | 2.2 (3) |

The care home use screening tools to identify hearing loss *

| Screening tools (%/n) | Less than 2 years | 2-5 years | 6-10 years | 10+ years |
| --- | --- | --- | --- | --- |
| Yes | 10.3 (8) | 10.7 (13) | 17.7 (11) | 23.2 (32) |
| No | 26.9 (21) | 45.9 (56) | 48.4 (30) | 55.8 (77) |
| Not Sure | 62.8 (49) | 43.4 (53) | 33.9 (21) | 21 (29) |

Residents hearing difficulties are recorded in their care plan

| Care plan (%/n) | Less than 2 years | 2-5 years | 6-10 years | 10+ years |
| --- | --- | --- | --- | --- |
| Yes | 88.5 (69) | 91 (111) | 91.9 (57) | 92.8 (128) |
| Sometimes | 5.1 (4) | 7.4 (9) | 6.5 (4) | 6.5 (9) |
| No | 1.3 (1) | 0 (0) | 0 (0) | 0 (0) |
| Not Sure | 5.1 (4) | 1.6 (2)) | 1.6 (1)) | 0.7 (1) |

The care home has dedicated public quiet areas *

| Access to quiet rooms (%/n) | Less than 2 years | 2-5 years | 6-10 years | 10+ years |
| --- | --- | --- | --- | --- |
| Yes | 69.2 (54) | 76.2 (93) | 88.7 (55) | 84.1 (116) |
| No | 12.8 (10) | 18 (22) | 9.7 (6) | 13.8 (19) |
| Not Sure | 17.9 (14) | 5.7 (7) | 1.6 (1) | 2.2 (3) |

I am confident in cleaning hearing aids

| Knowledge cleaning hearing aids (%/n) | Less than 2 years | 2-5 years | 6-10 years | 10 + years |
| --- | --- | --- | --- | --- |
| Strongly Agree | 14.1 (11) | 31.1 (38) | 17.7 (11) | 25.4 (35) |
| Agree | 50 (39) | 46.7 (57) | 61.3 (38) | 59.4 (82) |
| Disagree | 28.2 (22) | 18 (22) | 17.7 (11) | 13.8 (19) |
| Strongly Disagree | 7.7 (6) | 4.1 (5) | 3.2 (2) | 1.4 (2) |

Residents are willing to use their hearing aids provided

| Professional assessment (%/n) | Less than 2 years | 2-5 years | 6-10 years | 10+ years |
| --- | --- | --- | --- | --- |
| All | 26.9 (21) | 35.2 (43) | 24.2 (15) | 17.4 (24) |
| Some | 62.8 (49) | 58.2 (71) | 71 (44) | 71.7 (99) |
| None | 0.3 (1) | 0 (0) | 1.6 (1) | 2.9 (4) |

Hearing aids are checked regularly by care staff to see if they are working correctly *

| Aids checked regularly (%/n) | Less than 2 years | 2-5 years | 6-10 years | 10+ years |
| --- | --- | --- | --- | --- |
| Yes | 67.9 (53) | 86.9 (106) | 83.9 (52) | 90.6 (125) |
| No | 6.4 (5) | 3.3 (4) | 4.8 (3) | 5.8 (8) |
| Not Sure | 25.6 (20) | 9.8 (12) | 11.3 (7) | 3.6 (5) |

The majority of residents are able to take care of their own aid

| Knowledge cleaning hearing aids (%/n) | Less than 2 years | 2-5 years | 6-10 years | 10 + years |
| --- | --- | --- | --- | --- |
| Strongly Agree | 1.3 (1) | 4.9 (6) | 3.2 (2) | 0 (0) |
| Agree | 25.6 (20) | 18 (22) | 14.5 (9) | 16.7 (23) |
| Disagree | 60.3 (47) | 18 (22) | 66.1 (41) | 60.9 (84) |
| Strongly Disagree | 12.8 (10) | 13.9 (17) | 16.1 (10) | 22.5 (31) |

| Access to other aids (%/n) | Less than 2 years | 2-5 years | 6-10 years | 10+ years |
| --- | --- | --- | --- | --- |
| Yes | 12.8 (10) | 10.7 (13) | 12.9 (8) | 21 (29) |
| No | 25.6 (20) | 30.3 (27) | 30.6 (19) | 31.2 (43) |
| Not Sure | 34.6 (27) | 20.5 (25) | 16.1 (10) | 9.4 (13) |
| Only when they bring their own | 26.9 (21) | 38.5 (47) | 40.3 (25) | 38.4 (53) |

The residents have access to other hearing aids/ devices such as headphones and hearing loop systems

Family members recognise and assist with their relative hearing problems

| Family members assist (%/n) | Less than 2 years | 2-5 years | 6-10 years | 10+ years |
| --- | --- | --- | --- | --- |
| Yes | 37.2 (29) | 44.3 (54) | 38.7 (24) | 41.3 (57) |
| Sometimes | 59 (46) | 50.8 (62) | 58.1 (36) | 3.6 (5) |
| No | 3.8 (3) | 4.9 (6) | 3.2 (2) | 55.1 (76) |

All residents have annual hearing check-ups by professional audiologists *

| Professional assessment (%) | Less than 2 years | 2-5 years | 6-10 years | 10+ years |
| --- | --- | --- | --- | --- |
| Yes | 37.2 (29) | 54.9 (67) | 48.4 (30) | 44.2 (61) |
| No | 9 (7) | 17.2 (21) | 32.3 (20) | 37.7 (52) |
| Not Sure | 53.8 (42) | 27.9 (34) | 19.4 (12) | 18.1 (25) |

I am confident in communicating with residents with hearing difficulties

| Knowledge communicating (%/n) | Less than 2 years | 2-5 years | 6-10 years | 10+ years |
| --- | --- | --- | --- | --- |
| I am confident | 50 (39) | 68.9 (84) | 66.1 (41) | 65.2 (90) |
| I am confident but would like more information | 39.7 (31) | 27 (33) | 25.8 (16) | 29 (40) |
| I am not confident, but I know the basics | 6.4 (5) | 4.1 (5) | 6.5 (4) | 5.8 (8) |
| I am not confident at all | 3.8 (3) | 0 (0) | 1.6 (1) | 0 (0) |

I would like more information on how to better identify and manage hearing loss

| Want more information (%/n) | Less than 2 years | 2-5 years | 6-10 years | 10 + years |
| --- | --- | --- | --- | --- |
| Strongly Agree | 25.6 (20) | 31.1 (38) | 32.3 (20) | 21.7 (30) |
| Agree | 67.9 (53) | 54.1 (66) | 59.7 (37) | 68.1 (94) |
| Disagree | 3.8 (3) | 14.8 (18) | 8.1 (5) | 8.7 (12) |
| Strongly Disagree | 2.6 (2) | 0 (0) | 0 (0) | 1.4 (2) |

Vision

I know hot to tell the difference between the various vision problems

| Knowledge of vision problems (%/n) | Less than 2 years | 2-5 years | 6-10 years | 10 + years |
| --- | --- | --- | --- | --- |
| Strongly Agree | 3.8 (3) | 8.2 (10) | 4.8 (3) | 12.3 (17) |
| Agree | 39.7 (31) | 36.9 (45) | 46.8 (29) | 48.6 (67) |
| Disagree | 47.4 (37) | 46.7 (57) | 41.9 (26) | 34.8 (48) |
| Strongly Disagree | 9 (7) | 8.2 (10) | 6.5 (4) | 4.3 (6) |

I am confident in assessing whether a resident is poorly sighted

| Identifying vision problems (%/n) | Less than 2 years | 2-5 years | 6-10 years | 10 + years |
| --- | --- | --- | --- | --- |
| Strongly Agree | 3.8 (3) | 8.2 (10) | 4.8 (3) | 12.3 (17) |
| Agree | 66.7 (52) | 54.1 (66) | 64.5 (40) | 57.2 (79) |
| Disagree | 24.4 (19) | 28.7 (35) | 19.4 (12) | 24.6 (34) |
| Strongly Disagree | 9 (7) | 8.2 (10) | 6.5 (4) | 4.3 (6) |

I am confident in assessing whether a resident with cognitive difficulties is poorly sighted

| Identifying vision problems in cognitively impaired (%/n) | Less than 2 years | 2-5 years | 6-10 years | 10 + years |
| --- | --- | --- | --- | --- |
| Strongly Agree | 9 (7) | 15.6 (19) | 14.5 (9) | 16.7 (23) |
| Agree | 51.3 (40) | 49.2 (60) | 58.1 (36) | 51.4 (71) |
| Disagree | 34.6 (27) | 31.1 (38) | 25.8 (16) | 30.4 (42) |
| Strongly Disagree | 5.1 (4) | 4.1 (5) | 1.6 (1) | 1.4 (2) |

The care home use screening tools to identify vision loss *

| Screening tools (%/n) | Less than 2 years | 2-5 years | 6-10 years | 10+ years |
| --- | --- | --- | --- | --- |
| Yes | 20.5 (16) | 20.5 (25) | 24.2 (15) | 28.3 (39) |
| No | 21.8 (17) | 46.7 (57) | 50 (31) | 50.7 (70) |
| Not Sure | 57.7 (45) | 32.8 (40) | 25.8 (16) | 21 (29) |

Residents poor vision is recorded in their care plan

| Care plan (%/n) | Less than 2 years | 2-5 years | 6-10 years | 10+ years |
| --- | --- | --- | --- | --- |
| Yes | 85.9 (67) | 92.6 (113) | 98.4 (61) | 96.4 (133) |
| Sometimes | 5.1 (4) | 4.9 (6) | 1.6 (1) | 2.2 (3) |
| No | 1.3 (1) | 0 (0) | 0 (0) | 0.7 (1) |
| Not Sure | 7.7 (6) | 2.5 (3) | 0 (0) | 0.7 (1) |

The environment is well lit to cater for residents with poor vision

| Environment well lit (%/n) | Less than 2 years | 2-5 years | 6-10 years | 10+ years |
| --- | --- | --- | --- | --- |
| Yes | 79.5 (62) | 86.1 (105) | 93.5 (58) | 92.8 (128) |
| No | 5.1 (4) | 6.6 (8) | 3.2 (2) | 5.1 (7) |
| Not Sure | 15.4 (12) | 7.4 (9) | 3.2 (2) | 2.2 (3) |

Adaptations to the environment are used around the care home

| Environment adaptations (%/n) | Less than 2 years | 2-5 years | 6-10 years | 10+ years |
| --- | --- | --- | --- | --- |
| Yes | 57.7 (45) | 67.2 (82) | 72.6 (45) | 71.7 (99) |
| No | 20.5 (16) | 24.6 (30) | 17.7 (11) | 21.7 (30) |
| Not Sure | 21.8 (17) | 8.2 (10) | 9.7 (6) | 26.5 (9) |

I am confident cleaning vision aids such as residents glasses

| Knowledge cleaning glasses (%/n) | Less than 2 years | 2-5 years | 6-10 years | 10 + years |
| --- | --- | --- | --- | --- |
| Strongly Agree | 43.6 (34) | 60.7 (74) | 56.5 (35) | 87 (63) |
| Agree | 47.4 (37) | 38.5 (47) | 40.3 (25) | 35.5 (49) |
| Disagree | 7.7 (6) | 0 (0) | 1.6 (1) | 1.4 (2) |
| Strongly Disagree | 1.3 (1) | 0.8 (1) | 1.6 (1) | 0 (0) |

Residents are willing to use their glasses provided

| Residents willing (%/n) | Less than 2 years | 2-5 years | 6-10 years | 10 + years |
| --- | --- | --- | --- | --- |
| All | 26.9 (21) | 33.6 (41) | 30.6 (19) | 26.8 (37) |
| Some | 67.9 (53) | 63.1 (77) | 67.7 (42) | 69.6 (96) |
| Very few | 3.8 (3) | 3.3 (4) | 1.6 (1) | 13.6 (5) |
| None | 1.3 (1) | 0 (0) | 10 (0) | 0 (0) |

Residents glasses are cleaned regularly by care staff

| Glasses cleaned regularly (%/n) | Less than 2 years | 2-5 years | 6-10 years | 10+ years |
| --- | --- | --- | --- | --- |
| Yes | 83.3 (65) | 91.8 (112) | 91.9 (57) | 96.4 (133) |
| No | 6.4 (5) | 6.6 (8) | 4.8 (3) | 1.4 (2) |
| Not Sure | 8 (10.3) | 1.6 (2) | 3.2 (2) | 2.2 (3) |

Residents glasses are labelled with their names *

| Glasses labelled (%/n) | Less than 2 years | 2-5 years | 6-10 years | 10+ years |
| --- | --- | --- | --- | --- |
| Yes | 43.6 (34) | 54.1 (66) | 56.5 (35) | 43.5 (60) |
| No | 10.3 (8) | 8.2 (10) | 6.5 (4) | 5.1 (7) |
| Not Sure | 14.1 (11) | 1.6 (2) | 0 (0) | 2.2 (3) |
| Some | 32.1 (25) | 36.1 (44) | 37.1 (23) | 49.3 (68) |

Residents have access to other assistive devices such as large font books or magnifying glasses *

| Access to other aids (%/n) | Less than 2 years | 2-5 years | 6-10 years | 10+ years |
| --- | --- | --- | --- | --- |
| Yes | 28.2 (22) | 40.2 (49) | 53.2 (33) | 55.1 (76) |
| No | 7.7 (6) | 11.5 (14) | 9.7 (6) | 5.1 (7) |
| Not Sure | 35.9 (28) | 9 (11) | 4.8 (3) | 8.7 (12) |
| Only when they bring their own | 28.2 (22) | 39.3 (48) | 32.3 (20) | 31.2 (43) |

Family members recognise and assist with their relatives vision problems

| Family members assist (%/n) | Less than 2 years | 2-5 years | 6-10 years | 10 + years |
| --- | --- | --- | --- | --- |
| Strongly Agree | 7.7 (6) | 23 (28) | 14.5 (9) | 15.9 (22) |
| Agree | 83.3 (65) | 68.9 (84) | 75.8 (47) | 71 (99) |
| Disagree | 7.7 (6) | 7.4 (9) | 9.7 (6) | 12.3 (17) |
| Strongly Disagree | 1.3 (1) | 0.8 (1) | 0 (0) | 0 7 (1) |

All residents have annual vision check-ups by professional optometrists *

| Professional assessment (%/n) | Less than 2 years | 2-5 years | 6-10 years | 10+ years |
| --- | --- | --- | --- | --- |
| Yes | 61.5 (48) | 90.2 (110) | 90.3 (56) | 92 (127) |
| No | 1.3 (1) | 0.8 (1) | 3.2 (2) | 5.1 (7) |
| Not Sure | 37.2 (29) | 9 (11) | 6.5 (4) | 2.9 (4) |

I am confident in communicating with residents with poor vision

| Knowledge communicating (%/n) | Less than 2 years | 2-5 years | 6-10 years | 10+ years |
| --- | --- | --- | --- | --- |
| I am confident | 55.1 (43) | 76.2 (93) | 75.8 (47) | 71.7 (99) |
| I am confident but would like more information | 38.5 (30) | 20.5 (25) | 19.4 (12) | 26.1 (36) |
| I am not confident, but I know the basics | 5.1 (4) | 3.3 (4) | 4.8 (3) | 2.2 (3) |
| I am not confident at all | 1.3 (1) | 0 (0) | 0 (0) | 0 (0) |

I would like more information on how to better identify and manage vision loss

| Want more information (%/n) | Less than 2 years | 2-5 years | 6-10 years | 10 + years |
| --- | --- | --- | --- | --- |
| Strongly Agree | 30.8 (24) | 28.7 (35) | 24.2 (15) | 20.3 (28) |
| Agree | 59 (46) | 55.7 (68) | 62.9 (39) | 62.3 (86) |
| Disagree | 9 (7) | 13.9 (17) | 12.9 (8) | 15.9 (22) |
| Strongly Disagree | 21.3 (1) | 1.6 (2) | 0 (0) | 1.4 (2) |

**Care Home Type**

Hearing

I know how to tell the difference between the various hearing problems

| Knowledge of hearing problems (%/n) | Nursing | Residential | Nursing and Residential | Dementia Specific | Residential Dementia Specific | Other |
| --- | --- | --- | --- | --- | --- | --- |
| Strongly Agree | 17.1 (25) | 4.8 (7) | 7.7 (1) | 11.4 (5) | 26.7 (4) | 0 (0) |
| Agree | 55.3 (99) | 56.8 (83) | 84.6 (11) | 54.5 (24) | 33.3 (5) | 66.7 (2) |
| Disagree | 21.8 (39) | 25.3 (37) | 7.7 (1) | 31.8 (14) | 40 (6) | 33.3 (1) |
| Strongly disagree | 0.7 (1) | 6.8 (10) | 0 (0) | 2.3 (1) | 0 (0) | 0 (0) |

I am confident in assessing whether a resident has hearing difficulties

| Identifying hearing problems (%/n) | Nursing | Residential | Nursing and Residential | Dementia Specific | Residential Dementia Specific | Other |
| --- | --- | --- | --- | --- | --- | --- |
| Strongly Agree | 27.9 (50) | 24 (35) | 15.4 (2) | 15.9 (7) | 26.7 (4) | 33.3 (1) |
| Agree | 56.4 (101) | 63 (92) | 84.6 (11) | 61.4 (27) | 53.3 (8) | 33.3 (1) |
| Disagree | 12.3 (22) | 12.3 (18) | 0 (0) | 20.5 (9) | 20 (3) | 33.3 (1) |
| Strongly disagree | 3.4 (6) | 0.7 (1) | 0 (0) | 2.3 (1) | 0 (0) | 0 (0) |

I am confident in assessing whether a resident with cognitive difficulties has hearing difficulties

| Identifying hearing problems in cognitively impaired (%/n) | Nursing | Residential | Nursing and Residential | Dementia Specific | Residential Dementia Specific | Other |
| --- | --- | --- | --- | --- | --- | --- |
| Strongly agree | 20.1 (36) | 23.3 (34) | 23.1 (3) | 20.5 (9) | 20 (3) | 33.3 (1) |
| Agree | 57 (102) | 53.4 (78) | 61.5 (8) | 52.3 (23) | 53.3 (8) | 33.3 (1) |
| Disagree | 19.6 (35) | 21.9 (32) | 15.4 (2) | 25 (11) | 26.7 (4) | 33.3 (1) |
| Strongly disagree | 3.4 (6) | 1.4 (2) | 0 (0) | 2.3 (1) | 0 (0) | 0 (0) |

The care home use screening tools to identify hearing loss

| Screening tools (%/n) | Nursing | Residential | Nursing and Residential | Dementia Specific | Residential Dementia Specific | Other |
| --- | --- | --- | --- | --- | --- | --- |
| Yes | 23.5 (42) | 10.3 (15) | 23.1 (3) | 6.8 (3) | 6.7 (1) | 0 (0) |
| No | 44.1 (79) | 43.8 (64) | 46.2 (6) | 47.7 (21) | 73.3 (11) | 100 (3) |
| Not Sure | 32.4 (58) | 45.9 (67) | 30.8 (4) | 45.5 (20) | 20 (3) | 0 (0) |

Residents hearing difficulties are recorded in their care plan

| Care plans (%/n) | Nursing | Residential | Nursing and Residential | Dementia Specific | Residential Dementia Specific | Other |
| --- | --- | --- | --- | --- | --- | --- |
| Yes | 87.7 (157) | 93.8 (137) | 92.3 (12) | 93.2 (41) | 100 (15) | 100 (3) |
| Sometimes | 9.5 (17) | 4.1 (6) | 7.7 (1) | 4.5 (2) | 0 (0) | 0 (0) |
| No | 0.6 (1) | 0 (0) | 0 (0) | 0 (0) | 0 (0) | 0 (0) |
| Not Sure | 2.2 (4) | 2.1 (3) | 0 (0) | 2.3 (1) | 0 (0) | 0 (0) |

The care home has dedicated public quiet areas

| Access to quiet rooms (%/n) | Nursing | Residential | Nursing and Residential | Dementia Specific | Residential Dementia Specific | Other |
| --- | --- | --- | --- | --- | --- | --- |
| Yes | 78.8 (141) | 82.9 (121) | 84.6 (11) | 65.9 (29) | 93.3 (14) | 66.7 (2) |
| No | 15.1 (27) | 12.3 (18) | 7.7 (1) | 20.5 (9) | 6.7 (1) | 33.3 (1) |
| Not Sure | 6.1 (11) | 4.8 (7) | 7.7 (1) | 13.6 (6) | 0 (0) | 0 (0) |

I am confident in cleaning hearing aids

| Knowledge cleaning hearing aids (%/n) | Nursing | Residential | Nursing and Residential | Dementia Specific | Residential Dementia Specific | Other |
| --- | --- | --- | --- | --- | --- | --- |
| Strongly agree | 23.5 (42) | 24 (35) | 30.8 (4) | 20.5 (9) | 26.7 (4) | 33.3 (1) |
| Agree | 55.3 (99) | 56.2 (82) | 53.8 (7) | 47.7 (21) | 46.7 (7) | 0 (0) |
| Disagree | 17.9 (32) | 17.1 (25) | 7.7 (1) | 27.3 (12) | 13.3 (2) | 66.7 (2) |
| Strongly disagree | 3.4 (6) | 2.7 (4) | 7.7 (1) | 4.5 (2) | 13.3 (2) | 0 (0) |

Residents are willing to use their hearing aids provided *

| Residents willing to use aid (%/n) | Nursing | Residential | Nursing and Residential | Dementia Specific | Residential Dementia Specific | Other |
| --- | --- | --- | --- | --- | --- | --- |
| All | 26.3 (47) | 32.2 (47) | 15.4 (2) | 4.5 (2) | 33.3 (5) | 0 (0) |
| Some | 68.2 (122) | 65.1 (95) | 76.9 (10) | 54.5 (24) | 66.7 (10) | 66.7 (2) |
| Very few | 5.6 (10) | 2.1 (3) | 7.7 (1) | 29.5 (13) | 0 (0) | 33.3 (1) |
| None | 0 (0) | 0.7 (1) | 0 (0) | 11.4 (5) | 0 (0) | 0 (0) |

Hearing aids are checked regularly by care staff to see if they are working correctly *

| Aids checked regularly (%/n) | Nursing | Residential | Nursing and Residential | Dementia Specific | Residential Dementia Specific | Other |
| --- | --- | --- | --- | --- | --- | --- |
| Yes | 86.6 (155) | 81.5 (119) | 84.6 (11) | 86.4 (38) | 80 (12) | 33.2 (1) |
| No | 2.2 (4) | 5.5 (8) | 0 (0) | 11.4 (5) | 6.7 (1) | 66.7 (2) |
| Not Sure | 11.2 (20) | 13 (19) | 15.4 (2) | 2.3 (1) | 13.3 (2) | 0 (0) |

The majority of residents are able to take care of their own hearing aids *

| Residents care for own aid (%/n) | Nursing | Residential | Nursing and Residential | Dementia Specific | Residential Dementia Specific | Other |
| --- | --- | --- | --- | --- | --- | --- |
| Strongly Agree | 1.1 (2) | 4.8 (7) | 0 (0) | 0 (0) | 0 (0) | 0 (0) |
| Agree | 20.1 (36) | 21.9 (32) | 7.7 (1) | 2.3 (1) | 13.3 (2) | 66.7 (2) |
| Disagree | 62 (111) | 66.4 (97) | 76.9 (10) | 43.2 (19) | 80 (12) | 0 (0) |
| Strongly disagree | 16.8 (30) | 6.8 (10) | 15.4 (2) | 54.5 (24) | 6.7 (1) | 33.1 (1) |

The residents have access to other hearing aids

| Access to other aids (%/n) | Nursing | Residential | Nursing and Residential | Dementia Specific | Residential Dementia Specific | Other |
| --- | --- | --- | --- | --- | --- | --- |
| Yes | 15.1 (27) | 17.1 (25) | 30.8 (4) | 2.3 (1) | 6.7 (1) | 66.7 (2) |
| Only when residents bring their own | 40.2 (72) | 34.9 (51) | 46.2 (6) | 22.7 (10) | 40 (6) | 33.3 (1) |
| No | 29.6 (533) | 25.3 (37) | 0 (0) | 54.5 (24) | 33.3 (5) | 0 (0) |
| Not Sure | 15.1 (27) | 22.6 (33) | 23.1 (3) | 20.5 (9) | 20 (3) | 0 (0) |

Family members recognise and assist with their relatives hearing problems

| Family assist (%/n) | Nursing | Residential | Nursing and Residential | Dementia Specific | Residential Dementia Specific | Other |
| --- | --- | --- | --- | --- | --- | --- |
| Yes | 47.5 (85) | 35.6 (52) | 30.8 (4) | 34.1 (15) | 46.7 (7) | 0 (0) |
| Sometimes | 49.2 (88) | 58.9 (86) | 69.2 (9) | 61.4 (27) | 53.3 (8) |  |
| No | 3.4 (6) | 5.5 (8) | 0 (0) | 4.5 (2) | 0 (0) | 33.3 (1) |

All residents have annual hearing check-ups by professional audiologists

| Professional assessment (%/n) | Nursing | Residential | Nursing and Residential | Dementia Specific | Residential Dementia Specific | Other |
| --- | --- | --- | --- | --- | --- | --- |
| Yes | 57.5 (103) | 43.8 (64) | 38.5 (5) | 22.7 (10) | 33.3 (5) | 0 (0) |
| No | 20.7 (37) | 23.3 (34) | 23.1 (3) | 43.2 (19) | 40 (6) | 33.3 (1) |
| Not Sure | 21.8 (39) | 32.9 (48) | 38.5 (5) | 34.1 (15) | 26.7 (4) | 66.7 (2) |

I am confident in communicating with residents with hearing difficulties *

| Knowledge of how to communicate (%/n) | Nursing | Residential | Nursing and Residential | Dementia Specific | Residential Dementia Specific | Other |
| --- | --- | --- | --- | --- | --- | --- |
| I am confident | 60.3 (108) | 69.2 (101) | 61.5 (8) | 56.8 (25) | 73.3 (11) | 33.3 (1) |
| I am confident but would like more information | 34.6 (62) | 26 (38) | 23.1 (3) | 34.1 (15) | 13.3 (2) | 0 (0) |
| I am not confident but I know the basics | 4.5 (8) | 4.1 (6) | 15.4 (2) | 6.8 (3) | 13.3 (2) | 33.3 (1) |
| I am not confident at all | 0.6 (1) | 0.7 (1) | 0 (0) | 2.3 (1) | 0 (0) | 33.3 (1) |

I would like more information on how to better identify and manage hearing loss

| More information (%/n) | Nursing | Residential | Nursing and Residential | Dementia Specific | Residential Dementia Specific | Other |
| --- | --- | --- | --- | --- | --- | --- |
| Strongly Agree | 33.5 (60) | 20.5 (30) | 30.8 (4) | 22.7 (10) | 26.7 (4) | 0 (0) |
| Agree | 60.9 (109) | 65.8 (96) | 69.2 (9) | 54.5 (24) | 60 (9) | 100 (3) |
| Disagree | 5 (9) | 13.7 (20) | 0 (0) | 15.9 (7) | 13.3 (2) | 0 (0) |
| Strongly disagree | 0.6 (1) | 0 (0) | 0 (0) | 6.8 (3) | 0 (0) | 0 (0) |

Vision

I know how to tell the difference between the various vision problems

| Knowledge of vision problems (%/n) | Nursing | Residential | Nursing and Residential | Dementia Specific | Residential Dementia Specific | Other |
| --- | --- | --- | --- | --- | --- | --- |
| Strongly Agree | 7.3 (13) | 8.2 (12) | 15.4 (2) | 11.4 (5) | 6.7 (1) | 0 (0) |
| Agree | 48.6 (87) | 41.8 (61) | 53.8 (7) | 18.2 (8) | 53.3 (8) | 33.3 (1) |
| Disagree | 37.4 (67) | 43.8 (64) | 30.8 (4) | 59.1 (26) | 33.3 (5) | 66.7 (2) |
| Strongly disagree | 6.7 (12) | 6.2 (9) | 0 (0) | 11.4 (5) | 6.7 (1) | 0 (0) |

I am confident in assessing whether a resident is poorly sighted

| Identifying vision problems (%/n) | Nursing | Residential | Nursing and Residential | Dementia Specific | Residential Dementia Specific | Other |
| --- | --- | --- | --- | --- | --- | --- |
| Strongly Agree | 14 (25) | 14.4 (21) | 23.1 (3) | 9.1 (4) | 20 (3) | 0 (0) |
| Agree | 60.3 (108) | 58.2 (85) | 76.9 (10) | 52.3 (23) | 60 (9) | 66.7 (2) |
| Disagree | 24.6 (44) | 25.3 (37) | 0 (0) | 34.1 (15) | 20 (3) | 33.3 (1) |
| Strongly disagree | 1.1 (2) | 2.1 (3) | 0 (0) | 4.5 (2) | 0 (0) | 0 (0) |

I am confident in assessing whether a resident with cognitive difficulties is poorly sighted

| Identifying vision problems in cognitively impaired (%/n) | Nursing | Residential | Nursing and Residential | Dementia Specific | Residential Dementia Specific | Other |
| --- | --- | --- | --- | --- | --- | --- |
| Strongly Agree | 16.2 (29) | 11.6 (17) | 23.1 (3) | 15.9 (7) | 13.3 (2) | 0 (0) |
| Agree | 50.8 (91) | 56.2 (82) | 53.8 (7) | 36.4 (16) | 66.7 (10) | 33.3 (1) |
| Disagree | 30.2 (54) | 29.5 (43) | 23.1 (3) | 40.9 (18) | 20 (3) | 66.7 (2) |
| Strongly disagree | 2.8 (5) | 2.7 (4) | 0 (0) | 6.8 (3) | 0 (0) | 0 (0) |

The care home use screening tools to identify vision loss

| Screening tools (%/n) | Nursing | Residential | Nursing and Residential | Dementia Specific | Residential Dementia Specific | Other |
| --- | --- | --- | --- | --- | --- | --- |
| Yes | 29.6 (53) | 15.1 (22) | 38.5 (5) | 20.5 (9) | 33.3 (5) | 33.3 (1) |
| No | 43.6 (78) | 45.9 (67) | 38.5 (5) | 40.9 (18) | 40 (6) | 33.3 (1) |
| Not Sure | 26.8 (48) | 39 (57) | 23.1 (3) | 38.6 (17) | 26.7 (4) | 33.3 (1) |

Residents poor vision is recorded in their care plan

| Care plan (%/n) | Nursing | Residential | Nursing and Residential | Dementia Specific | Residential Dementia Specific | Other |
| --- | --- | --- | --- | --- | --- | --- |
| Yes | 93.9 (168) | 91.8 (134) | 100 (13) | 93.2 (41) | 100 (15) | 100 (3) |
| Sometimes | 4.5 (8) | 2.7 (4) | 0 (0) | 4.5 (2) | 0 (0) | 0 (0) |
| No | 0.6 (1) | 0.7 (1) | 0 (0) | 0 (0) | 0 (0) | 0 (0) |
| Not Sure | 1.1 (2) | 4.8 (7) | 0 (0) | 2.3 (1) | 0 (0) | 0 (0) |

The care home is well lit to cater for residents with poor vision

| Environment well lit (%/n) | Nursing | Residential | Nursing and Residential | Dementia Specific | Residential Dementia Specific | Other |
| --- | --- | --- | --- | --- | --- | --- |
| Yes | 88.8 (159) | 85.6 (125) | 92.3 (12) | 95.5 (42) | 80 (12) | 100 (3) |
| No | 4.5 (8) | 7.5 (11) | 0 (0) | 0 (0) | 13.3 (2) | 0 (0) |
| Not Sure | 6.7 (12) | 6.8 (10) | 7.7 (1) | 4.5 (2) | 6.7 (1) | 0 (0) |

Adaptations to the environment are used around the care home

| Environment adaptations (%/n) | Nursing | Residential | Nursing and Residential | Dementia Specific | Residential Dementia Specific | Other |
| --- | --- | --- | --- | --- | --- | --- |
| Yes | 64.8 (116) | 74 (108) | 84.6 (11) | 52.3 (23) | 73.3 (11) | 66.7 (2) |
| No | 23.5 (42) | 15.1 (22) | 7.7 (1) | 43.2 (19) | 20 (3) | 0 (0) |
| Not Sure | 11.7 (21) | 11 (16) | 7.7 (1) | 4.5 (2) | 6.7 (1) | 33.3 (1) |

I am confident in cleaning vision aids such as residents glasses

| Knowledge cleaning glasses (%/n) | Nursing | Residential | Nursing and Residential | Dementia Specific | Residential Dementia Specific | Other |
| --- | --- | --- | --- | --- | --- | --- |
| Strongly Agree | 54.7 (98) | 56.8 (83) | 53.8 (7) | 61.4 (27) | 86.7 (13) | 66.7 (2) |
| Agree | 43 (77) | 39.7 (58) | 46.2 (6) | 34.1 (15) | 13.3 (2) | 0 (0) |
| Disagree | 1.7 (3) | 3.4 (5) | 0 (0) | 0 (0) | 0 (0) | 33.3 (1) |
| Strongly disagree | 0.6 (1) | 0 (0) | 0 (0) | 4.5 (2) | 0 (0) | 0 (0) |

Residents are willing to use their glasses provided *

| Residents willing to use glasses (%) | Nursing | Residential | Nursing and Residential | Dementia Specific | Residential Dementia Specific | Other |
| --- | --- | --- | --- | --- | --- | --- |
| All | 35.2 (63) | 32.2 (47) | 15.4 (2) | 4.5 (2) | 20 (3) | 33.3 (1) |
| Some | 63.7 (114) | 67.1 (98) | 76.9 (10) | 72.7 (32) | 80 (12) | 66.7 (2) |
| Very few | 1.1 (2) | 0.7 (1) | 7.7 (1) | 20.5 (9) | 0 (0) | 0 (0) |
| None | 0 (0) | 0 (0) | 0 (0) | 2.3 (1) | 0 (0) | 0 (0) |

Residents glasses are cleaned regularly by care staff

| Glasses cleaned regularly (%/n) | Nursing | Residential | Nursing and Residential | Dementia Specific | Residential Dementia Specific | Other |
| --- | --- | --- | --- | --- | --- | --- |
| Yes | 91.1 (163) | 91.1 (133) | 92.3 (12) | 95.5 (42) | 100 (15) | 66.7 (2) |
| No | 3.4 (6) | 6.2 (9) | 7.7 (1) | 2.3 (1) | 0 (0) | 33.3 (1) |
| Not Sure | 5.6 (10) | 2.7 (4) | 0 (0) | 2.3 (1) | 0 (0) | 0 (0) |

Residents glasses are labelled with their names *

| Glasses labelled (%/n) | Nursing | Residential | Nursing and Residential | Dementia Specific | Residential Dementia Specific | Other |
| --- | --- | --- | --- | --- | --- | --- |
| Yes | 53.1 (95) | 13.7 (20) | 38.5 (5) | 65.9 (29) | 0 (0) | 66.7 (2) |
| Some | 37.4 (67) | 2.7 (4) | 7.7 (1) | 31.8 (14) | 0 (0) | 0 (0) |
| No | 3.4 (6) | 36.3 (53) | 53.8 (7) | 0 (0) | 80 (12) | 33.3 (1) |
| Not Sure | 6.1 (11) | 47.3 (69) | 0 (0) | 2.3 (1) | 20 (3) | 0 (0) |

Residents have access to other assistive devices

| Access to other aids (%/n) | Nursing | Residential | Nursing and Residential | Dementia Specific | Residential Dementia Specific | Other |
| --- | --- | --- | --- | --- | --- | --- |
| Yes | 46.9 (84) | 52.7 (77) | 38.5 (5) | 18.2 (8) | 33.3 (5) | 33.3 (1) |
| Only when residents bring their own | 33.5 (60) | 28.1 (41) | 38.5 (5) | 40.9 (18) | 53.3 (8) | 33.3 (1) |
| No | 6.7 (12) | 6.8 (10) | 0 (0) | 20.5 (9) | 6.7 (1) | 33.3 (1) |
| Not Sure | 12.8 (23) | 12.3 (18) | 23.1 (3) | 20.5 (9) | 6.7 (1) | 0 (0) |

Family members recognise and assist with their relatives vision problems

| Family members assist (%/n) | Nursing | Residential | Nursing and Residential | Dementia Specific | Residential Dementia Specific | Other |
| --- | --- | --- | --- | --- | --- | --- |
| Strongly Agree | 14.5 (26) | 18.5 (27) | 23.1 (3) | 13.6 (6) | 20 (3) | 0 (0) |
| Agree | 74.9 (134) | 73.3 (107) | 61.5 (8) | 77.3 (34) | 53.3 (8) | 100 (3) |
| Disagree | 10.1 (18) | 7.5 (11) | 15.4 (2) | 6.8 (3) | 26.7 (4) | 0 (0) |
| Strongly disagree | 0.6 (1) | 0.7 (1) | 0 (0) | 42.3 (1) | 0 (0) | 0 (0) |

All residents have annual vision check-ups by professional optometrists

| Professional assessment (%/n) | Nursing | Residential | Nursing and Residential | Dementia Specific | Residential Dementia Specific | Other |
| --- | --- | --- | --- | --- | --- | --- |
| Yes | 86.6 (155) | 82.2 (120) | 100 (13) | 86.4 (38) | 93.3 (14) | 33.3 (1) |
| No | 2.2 (4) | 3.4 (5) | 0 (0) | 2.3 (1) | 6.7 (1) | 0 (0) |
| Not Sure | 11.2 (20) | 14.4 (21) | 0 (0) | 11.4 (5) | 0 (0) | 66.7 (2) |

I am confident in communicating with residents with poor vision

| Knowledge of how to communicate (%/n) | Nursing | Residential | Nursing and Residential | Dementia Specific | Residential Dementia Specific | Other |
| --- | --- | --- | --- | --- | --- | --- |
| I am confident | 65.4 (117) | 77.4 (113) | 69.2 (9) | 65.9 (29) | 80 (12) | 66.7 (2) |
| I am confident but would like more information | 30.2 (54) | 20.5 (30) | 23.1 (3) | 29.5 (13) | 20 (3) | 0 (0) |
| I am not confident but I know the basics | 4.5 (8) | 42.1 (3) | 7.7 (1) | 2.3 (1) | 0 (0) | 33.3 (1) |
| I am not confident at all | 0 (0) | 0 (0) | 0 (0) | 2.3 (1) | 0 (0) | 0 (0) |

I would like more information on how to better identify and manage vision loss

| More information (%/n) | Nursing | Residential | Nursing and Residential | Dementia Specific | Residential Dementia Specific | Other |
| --- | --- | --- | --- | --- | --- | --- |
| Strongly Agree | 29.1 (52)) | 22.6 (33) | 23.1 (3) | 25 (11) | 20 (3) | 0 (0) |
| Agree | 62.6 (112) | 59.6 (87) | 61.5 (8) | 47.7 (21) | 60 (9) | 66.7 (2) |
| Disagree | 7.8 (14) | 16.4 (24) | 15.4 (2) | 22.7 (10) | 20 (3) | 33.3 (1) |
| Strongly disagree | 0.6 (1) | 1.4 (2) | 0 (0) | 4.5 (2) | 0 (0) | 0 (0) |

**Size of care home**

Hearing

I know how to tell the difference between the various hearing problems

| Knowledge of hearing problems (%/n) | 0-30 | 31-60 | 61+ |
| --- | --- | --- | --- |
| Strongly agree | 18.6 (22) | 21.8 (31) | 12.1 (17) |
| Agree | 49.2 (58) | 54.2 (77) | 63.6 (89) |
| Disagree | 30.5 (36) | 21.8 (31) | 22.1 (31) |
| Strongly disagree | 1.7 (2) | 2.1 (3) | 2.1 (3) |

I am confident in assessing whether a resident has hearing difficulties

| Identifying hearing problems (%/n) | 0-30 | 31-60 | 61+ |
| --- | --- | --- | --- |
| Strongly agree | 25.4 (30) | 31.7 (45) | 17.1 (24) |
| Agree | 57.6 (68) | 57 (81) | 65 (91) |
| Disagree | 15.3 (18) | 10.6 (15) | 14.3 (20) |
| Strongly disagree | 1.7 (2) | 0.7 (1) | 3.6 (5) |

I am confident in assessing whether a resident with cognitive difficulties has hearing difficulties

| Identifying hearing problems in cognitively impaired (%/n) | 0-30 | 31-60 | 61+ |
| --- | --- | --- | --- |
| Strongly agree | 19.5 (23) | 28.2 (40) | 16.4 (23) |
| Agree | 48.3 (57) | 52.1 (74) | 63.6 (89) |
| Disagree | 29.7 (35) | 19 (27) | 16.4 (23) |
| Strongly disagree | 2.5 (3) | 0.7 (1) | 3.6 (5) |

The care home use screening tools to identify hearing loss

| Screening tools (%/n) | 0-30 | 31-60 | 61+ |
| --- | --- | --- | --- |
| Yes | 8.5 (10) | 17.6 (25) | 20.7 (29) |
| No | 51.7 (61) | 45.1 (64) | 42.1 (59) |
| Not Sure | 39.8 (47) | 37.3 (53) | 37.1 (52) |

Residents hearing difficulties are recorded in their care plan

| Care plan (%/n) | 0-30 | 31-60 | 61+ |
| --- | --- | --- | --- |
| Yes | 93.2 (110) | 90.1 (128) | 90.7 (127) |
| Sometimes | 5.9 (7) | 4.9 (7) | 8.6 (12) |
| No | 0 (0) | 0.7 (1) | 0 (0) |
| Not Sure | 0.8 (1) | 4.2 (6) | 0.7 (1) |

The care home has dedicated public quiet areas

| Quiet environment (%/n) | 0-30 | 31-60 | 61+ |
| --- | --- | --- | --- |
| Yes | 78 (92) | 85.2 (12) | 75 (105) |
| No | 18.6 (22) | 8.5 (12) | 16.4 (23) |
| Not Sure | 3.4 (4) | 6.3 (9) | 8.6 (12) |

I am confident in cleaning hearing aids

| Knowledge of cleaning aids (%/n) | 0-30 | 31-60 | 61+ |
| --- | --- | --- | --- |
| Strongly agree | 24.6 (29) | 24.6 (35) | 22.1 (31) |
| Agree | 55.9 (66) | 52.8 (75) | 53.6 (75) |
| Disagree | 14.4 (17) | 20.4 (29) | 20 (28) |
| Strongly disagree | 5.1 (6) | 2.1 (3) | 4.3 (6) |

Residents are willing to use their hearing aids provided

| Residents willing (%/n) | 0-30 | 31-60 | 61+ |
| --- | --- | --- | --- |
| All | 31.4 (37) | 27.5 (39) | 19.3 (27) |
| Some | 55.1 (65) | 69 (98) | 71.4 (100) |
| Very few | 9.3 (11) | 3.5 (5) | 8.6 (12) |
| None | 4.2 (5) | 0 (0) | 0.7 (1) |

Hearing aids are checked regularly by care staff to see if they are working correctly

| Aids checked regularly (%/n) | 0-30 | 31-60 | 61+ |
| --- | --- | --- | --- |
| Yes | 85.6 (101) | 85.2 (121) | 81.4 (114) |
| No | 5.1 (6) | 4.9 (7) | 5 (7) |
| Not Sure | 9.3 (11) | 9.9 (14) | 13.6 (19) |

The majority of residents are able to take care of their own hearing aids

| Aids cared for by resident (%/n) | 0-30 | 31-60 | 61+ |
| --- | --- | --- | --- |
| Strongly agree | 0.8 (1) | 1.4 (2) | 4.3 (6) |
| Agree | 16.9 (20) | 19 (27) | 19.3 (27) |
| Disagree | 66.1 (78) | 61.3 (87) | 60 (84) |
| Strongly disagree | 16.1 (19) | 18.3 (26) | 16.4 (23) |

The residents have access to other hearing aids/devices

| Access to other aids (%/n) | 0-30 | 31-60 | 61+ |
| --- | --- | --- | --- |
| Yes | 16.1 (19) | 17.6 (25) | 11.4 (16) |
| Only when residents bring their own | 33.9 (40) | 38.7 (55) | 36.4 (51) |
| No | 35.6 (42) | 23.9 (34) | 30.7 (43) |
| Not Sure | 14.4 (17) | 19.7 (28) | 21.4 (30) |

Family members recognise and assist with their relatives hearing problems

| Family members assist (%/n) | 0-30 | 31-60 | 61+ |
| --- | --- | --- | --- |
| Yes | 31.4 (37) | 45.1 (64) | 45 (63) |
| Sometimes | 59.3 (70) | 53.5 (76) | 52.9 (74) |
| No | 9.3 (11) | 1.4 (2) | 2.1 (3) |

All residents have annual hearing check-ups by professional audiologists

| Professional assessments (%/n) | 0-30 | 31-60 | 61+ |
| --- | --- | --- | --- |
| Yes | 44.9 (53) | 46.5 (66) | 48.6 (68) |
| No | 35.6 (42) | 21.8 (31) | 19.3 (27) |
| Not sure | 19.5 (23) | 31.7 (45) | 32.1 (45) |

I am confident in communicating with residents with hearing difficulties

| Communicating (%/n) | 0-30 | 31-60 | 61+ |
| --- | --- | --- | --- |
| I am confident | 65.3 (77) | 59.2 (84) | 66.4 (93) |
| I am confident but would like more information | 28 (33) | 35.2 (50) | 26.4 (37) |
| I am not confident but I know the basics | 5.1 (6) | 5.6 (8) | 5.7 (8) |
| I am not confident at all | 1.7 (2) | 0 (0) | 1.4 (2) |

I would like more information on how to better identify and manage hearing loss

| Want more information (%/n) | 0-30 | 31-60 | 61+ |
| --- | --- | --- | --- |
| Strongly agree | 20.3 (24) | 28.2 (40) | 31.4 (44) |
| Agree | 66.1 (78) | 64.8 (92) | 57.1 (80) |
| Disagree | 11.9 (14) | 7 (10) | 10 (14) |
| Strongly disagree | 1.7 (2) | 0 (0) | 1.4 (2) |

Vision

I know how to tell the difference between the various vision problems

| Knowledge of vision problems (%/n) | 0-30 | 31-60 | 61+ |
| --- | --- | --- | --- |
| Strongly agree | 2.5 (3) | 12 (17) | 9.3 (13) |
| Agree | 44.9 (53) | 39.4 (56) | 45 (63) |
| Disagree | 47.5 (56) | 41.5 (59) | 37.9 (52) |
| Strongly disagree | 5.1 (6) | 7 (10) | 7.9 (11) |

I am confident in assessing whether a resident is poorly sighted

| Identifying vision problems (%/n) | 0-30 | 31-60 | 61+ |
| --- | --- | --- | --- |
| Strongly agree | 11 (13) | 16.9 (24) | 13.6 (19) |
| Agree | 61.9 (73) | 59.2 (84) | 57.1 (80) |
| Disagree | 25.4 (30) | 23.9 (34) | 25.7 (36) |
| Strongly disagree | 1.7 (2) | 0 (0) | 3.6 (5) |

I am confident in assessing whether a resident with cognitive difficulties is poorly sighted

| Identifying vision problems in cognitively impaired (%/n) | 0-30 | 31-60 | 61+ |
| --- | --- | --- | --- |
| Strongly agree | 8.5 (10) | 19 (27) | 15 (21) |
| Agree | 57.6 (68) | 50.7 (72) | 47.9 (67) |
| Disagree | 31.4 (37) | 28.9 (41) | 32.1 (45) |
| Strongly disagree | 2.5 (3) | 1.4 (2) | 5 (7) |

The care home use screening tools to identify vision loss

| Screening tools (%/n) | 0-30 | 31-60 | 61+ |
| --- | --- | --- | --- |
| Yes | 19.5 (23) | 26.8 (38) | 24.3 (34) |
| No | 51.7 (61) | 38.7 (55) | 42.1 (59) |
| Not sure | 28.8 (34) | 34.5 (49) | 33.6 (47) |

Residents poor vision is recorded in their care plan

| Care plan (%/n) | 0-30 | 31-60 | 61+ |
| --- | --- | --- | --- |
| Yes | 92.4 (109) | 91.5 (130) | 96.4 (135) |
| Sometimes | 4.2 (5) | 4.9 (7) | 1.4 (2) |
| No | 0.8 (1) | 0.7 (1) | 0 (0) |
| Not sure | 2.5 (3) | 2.8 (4) | 2.1 (3) |

The care home is well lit to cater for residents with poor vision

| Environment well lit (%/n) | 0-30 | 31-60 | 61+ |
| --- | --- | --- | --- |
| Yes | 79.7 (94) | 32.2 (47) | 91.4 (128) |
| No | 11 (13) | 4.2 (6) | 1.4 (2) |
| Not Sure | 9.3 (11) | 3.5 (5) | 7.1 (10) |

Adaptations to the environment are used around the care home

| Environment adaptations (%/n) | 0-30 | 31-60 | 61+ |
| --- | --- | --- | --- |
| Yes | 70.3 (83) | 66.2 (94) | 67.1 (94) |
| No | 22 (26) | 23.9 (34) | 19.3 (27) |
| Not Sure | 97.6 (9) | 9.9 (14) | 13.6 (19) |

I am confident in cleaning vision aids such as residents glasses

| Knowledge of cleaning glasses (%/n) | 0-30 | 31-60 | 61+ |
| --- | --- | --- | --- |
| Strongly agree | 65.3 (77) | 58.5 (83) | 50 (70) |
| Agree | 33.1 (39) | 38.7 (55) | 45.7 (64) |
| Disagree | 0.8 (1) | 2.8 (4) | 2.9 (4) |
| Strongly disagree | 0.8 (1) | 0 (0) | 1.4 (2) |

Residents are willing to use their glasses provided

| Residents willing (%/n) | 0-30 | 31-60 | 61+ |
| --- | --- | --- | --- |
| All | 34.7 (41) | 28.2 (40) | 26.4 (37) |
| Some | 61 (72) | 71.1 (101) | 67.9 (95) |
| Very Few | 3.4 (4) | 0.7 (1) | 5.7 (8) |
| None | 0.8 (1) | 0 (0) | 0 (0) |

Residents glasses are cleaned regularly by care staff

| Glasses cleaned regularly (%/n) | 0-30 | 31-60 | 61+ |
| --- | --- | --- | --- |
| Yes | 93.2 (11) | 92.3 (131) | 90 (126) |
| No | 4.2 (5) | 3.5 (5) | 5.7 (8) |
| Not Sure | 2.5 (3) | 4.2 (6) | 4.3 (6) |

Residents glasses are labelled with their names

| Glasses labelled (%/n) | 0-30 | 31-60 | 61+ |
| --- | --- | --- | --- |
| Yes | 52.5 (62) | 40.1 (57) | 54.3 (76) |
| Some | 38.1 (45) | 50 (71) | 31.4 (44) |
| No | 6.8 (8) | 5.6 (8) | 9.3 (13) |
| Not Sure | 2.5 (3) | 4.2 (6) | 5 (7) |

Residents have access to other assistive devices

| Access to other aids (%/n) | 0-30 | 31-60 | 61+ |
| --- | --- | --- | --- |
| Yes | 46.6 (55) | 52.1 (74) | 36.4 (51) |
| Only when residents bring their own | 35.6 (42) | 28.2 (40) | 36.4 (51) |
| No | 8.5 (10) | 4.2 (6) | 12.1 (17) |
| Not Sure | 9.3 (11) | 15.5 (22) | 15 (21) |

Family members recognise and assist with their relatives vision problems

| Family assist (%/n) | 0-30 | 31-60 | 61+ |
| --- | --- | --- | --- |
| Strongly agree | 16.1 (19) | 19.7 (28) | 12.9 (18) |
| Agree | 66.1 (78) | 72.5 (103) | 80.7 (113) |
| Disagree | 16.1 (19) | 7.7 (11) | 5.7 (8) |
| Strongly disagree | 1.7 (2) | 0 (0) | 0.7 (1) |

All residents have annual vision check-ups by professional optometrists

| Professional assessments (%/n) | 0-30 | 31-60 | 61+ |
| --- | --- | --- | --- |
| Yes | 91.5 (108) | 81.7 (116) | 83.6 (117) |
| No | 1.7 (2) | 4.9 (7) | 1.4 (2) |
| Not Sure | 6.8 (8) | 13.4 (19) | 15 (21) |

I am confident in communicating with residents with poor vision

| Communicating (%/n) | 0-30 | 31-60 | 61+ |
| --- | --- | --- | --- |
| I am confident | 74.6 (88) | 62 (88) | 75.7 (106) |
| I am confident but would like more information | 27.2 (28) | 33.1 (47) | 20 (28) |
| I am not confident but I know the basics | 0.8 (1) | 4.9 (7) | 4.3 (6) |
| I am not confident at all | 0.8 (1) | 0 (0) | 0 (0) |

I would like more information on how to better identify and manage vision loss

| Want more information (%/n) | 0-30 | 31-60 | 61+ |
| --- | --- | --- | --- |
| Strongly agree | 27.1 (32) | 28.2 (40) | 21.4 (30) |
| Agree | 52.5 (62) | 61.3 (87) | 64.3 (90) |
| Disagree | 17.8 (21) | 10.6 (15) | 12.9 (18) |
| Strongly disagree | 2.5 (3) | 0 (0) | 1.4 (2) |
